# Hybrid Neural Network-Based PDR with Multi-Layer Heading Correction Across Smartphone Carrying Modes

**DOI:** 10.3390/s26082421

**Published:** 2026-04-15

**Authors:** Junhua Ye, Anzhe Ye, Ahmed Mansour, Shusu Qiu, Zhenzhen Li, Xuanyu Qu

**Affiliations:** 1 School of Environmental and Resource Science, Zhejiang Agriculture and Forestry University, Hangzhou 311300, China; yejunhua2020@zafu.edu.cn (J.Y.); 2024chqss@stu.zafu.edu.cn (S.Q.); lizhenzhen2025@zafu.edu.cn (Z.L.); 2 Public Works Department, Faculty of Engineering, Cairo University, Giza 12613, Egypt; ahmed.m.mostafa@connect.polyu.hk; 3 Department of Land Surveying and Geo-Informatics, The Hong Kong Polytechnic University, Hong Kong; xuany.qu@connect.polyu.hk

**Keywords:** smartphone localization, CNN-LSTM, multi-carrying mode, pedestrian navigation, heading drift correction

## Abstract

Traditional pedestrian inertial navigation (PDR) algorithms usually assume that the carrying mode of a smartphone is fixed and remains horizontal, while ignoring the significant impact of dynamic changes in the carrying mode on heading estimation, which is the core element of PDR algorithms. In practical application scenarios, pedestrians often change their way of carrying smart terminals (e.g., calling) according to their needs, corresponding to the difference in the heading estimation method; especially when the mode is switched, it will cause a sudden change in heading, which will lead to a significant increase in the localization error if it cannot be corrected in time. Existing smart terminal carrying mode recognition methods that rely on traditional machine learning or set thresholds have poor robustness; lack of universality, especially weak diagnostic ability for mutation; and can not effectively reduce the heading error. Based on these practical problems, this paper innovatively proposes a PDR framework that tries to overcome these limitations. Based on this research purpose, firstly, this paper classifies four types of common carrying modes based on practical applications and designs a CNN-LSTM hybrid model, which can classify the four common carrying modes in near real-time, with a recognition accuracy as high as 99.68%. Secondly, based on the mode recognition results, a multi-layer heading correction strategy is introduced: (1) introducing a quaternion-based universal filter (VQF) algorithm to realize the accurate estimation of initial heading; (2) designing an algorithm to accurately detect the mode switching point and developing an adaptive offset correction algorithm to realize the dynamic compensation of heading in the process of mode switching to reduce the impact of sudden changes; and (3) considering the motion characteristics of pedestrians walking in a straight line segment where lateral displacement tends to be close to zero. This study designs a heading optimization method with lateral displacement constraints to further inhibit the drifting of the heading caused by the slight swaying of the smart terminal. In this study, two validation experiments are carried out in two different environment—an indoor corridor and a tree shelter—and the results show that based on the proposed multi-layer heading optimization strategy, the average heading error of the system is lower than 1.5°, the cumulative positioning error is lower than 1% of the walking distance, and the root mean square error of the checkpoints is lower than 2 m, which significantly reduces the positioning error and shows the effectiveness of the framework in complex environments.

## 1. Introduction

Indoor positioning has become a critical enabler for location-based services, navigation in Global Navigation Satellite System (GNSS)-denied environments, and context-aware mobile applications. Unlike outdoor GPS, reliable indoor positioning remains challenging due to the unavailability or distortion of satellite signals inside buildings. Researchers have explored diverse technologies for indoor localization, from wireless signals (WiFi, Bluetooth, Ultra-Wideband (UWB)) to visual Simultaneous Localization and Mapping (SLAM) and magnetic fingerprinting, but each has trade-offs in infrastructure requirements and reliability. In this landscape, pedestrian dead reckoning (PDR) based on inertial sensors stands out as an attractive infrastructure-free approach. Modern smartphones are equipped with micro-electromechanical inertial measurement units (IMUs), making PDR a ubiquitous solution for tracking a user’s motion using only on-board sensors. However, smartphone PDR is notorious for accumulated errors (drift) and sensitivity to how the device is carried.

In a typical PDR system, the user’s step-wise movements are inferred by detecting steps, estimating step lengths, and updating position based on the heading direction of travel. Heading estimation is arguably the most crucial and error-prone component, as even a small orientation error can cause the estimated trajectory to deviate significantly over distance. Without external reference, orientation errors from low-cost gyroscopes accumulate as heading drift. For instance, a yaw rate bias of just a few degrees per minute can lead to tens of meters of position error over a short walk. Consumer-grade smartphone IMUs suffer from bias and noise, so a straightforward integration of gyro readings is insufficient for long-term navigation stability. Magnetometers can provide an absolute heading reference by sensing the Earth’s field, but indoor magnetic disturbances often render compass readings unreliable. Thus, heading drift remains a fundamental limitation of inertial navigation on phones, necessitating clever drift correction or compensation strategies.

Compounding the problem, smartphone carrying mode variability greatly impacts PDR performance. A user might hold the phone in their hand, swing it by the side, place it in a pocket or bag, or even switch between these modes during a journey. Each mode alters the orientation and dynamics of the IMU relative to the user’s direction of travel. For example, when a phone is in a pocket, the sensor axes are misaligned with the walking direction, and step impacts have different signatures than when the phone is handheld. Classical PDR algorithms struggle in such scenarios because they often assume a fixed relationship between device frame and user motion. If unaccounted for, mode changes can lead to errors in step detection (e.g., lower acceleration peaks in a pocket) and misestimated headings (due to arbitrary device orientation). Recent studies have highlighted the importance of context awareness in PDR: recognizing the phone’s placement or the user’s activity to adjust the tracking algorithm. Traditional solutions have included training classifiers to detect the carrying mode or activity and then switching parameter sets accordingly. For instance, Khalili et al. use decision trees and Support Vector Machines (SVMs) to identify modes like texting vs. calling vs. pocket, and adapt step length models for each case, yielding improved accuracy over one-size-fits-all PDR. Still, handling abrupt transitions between modes and ensuring one algorithm works robustly for all poses remains an open challenge. A truly robust inertial navigation system must be pose-invariant, or capable of dynamically recalibrating when the device orientation changes [1].

Early approaches to mitigate heading drift and orientation issues relied on sensor fusion and heuristic calibration. One classical solution is the foot-mounted INS: strapping the IMU to the user’s shoe. With each footstep, the foot has a zero-velocity phase when it is on the ground; zero-velocity updates (ZUPTs) can be applied to correct velocity and drift errors on every step. This approach, combined with Kalman filtering, dramatically reduces drift and has been shown to constrain heading error as well. Pioneering work by Foxlin (2005) [2] demonstrated accurate pedestrian tracking with a shoe-mounted IMU by resetting velocity at foot stance and even estimating heading via foot orientation at each step. Likewise, Stirling et al. (2005) [3] introduced methods for heading estimation using shoe sensors that achieved excellent stability over time. The drawback, of course, is the requirement of external hardware on the foot, which is impractical for most smartphone users. Another approach is to leverage maps or known landmarks: for example, using the building’s corridor alignment as a reference to correct heading drift [4]. Such map-aiding or building heading alignment can constrain long-term drift, but again presumes external information (digital floor plans or environmental knowledge). Magnetometer fusion in an AHRS (attitude and heading reference system) is the standard smartphone-based method to bind heading to a global reference. Filters like Kalman or complementary filters blend gyro and magnetometer readings to output an absolute heading. However, when the magnetometer is distorted (near ferrous materials or electrical equipment), these filters can do more harm than good, leading to sudden heading jumps or false stabilization [5]. Researchers have proposed techniques to selectively trust magnetometer data only when it appears “healthy”, or to detect and ignore magnetic anomalies, but no solution is foolproof in all indoor settings. Some works incorporate additional sensors like barometers for stair detection, or even cameras: for example, Ye et al. (2025) [6] fuse inertial data with visual cues from the phone’s camera to correct heading by recognizing known floor patterns, significantly reducing drift during straight-line walking. While effective, camera-based methods impose a high computational load and assume the presence of visually discernible features. In summary, classical sensor fusion PDR methods improve accuracy via hardware (foot sensors, visual aids, UWB beacons) or assumptions about the environment, but these add cost or complexity. Generally speaking, the key ambition is the development of a smartphone-only navigation system capable of maintaining robust tracking performance under diverse real-world conditions, independent of user habits, device pose, or external reference signals.

In recent years, data-driven and AI-based approaches have surged to tackle the limitations of traditional PDR. The growing availability of sensor data and advances in deep learning have enabled researchers to learn inertial navigation directly from data, rather than hand-crafting every component [7]. Deep learning models can theoretically capture complex user motion characteristics, sensor biases, and context effects automatically through training. One line of work uses neural networks to estimate velocity or displacement increments from a window of IMU readings, effectively learning the PDR step without explicit step detection. For example, Yan et al. (2018) [8] introduced RIDI, an early data-driven method where a neural network learned to output velocity corrections to assist double-integration of accelerometer data. This helped constrain drift by compensating for biases in the acceleration signal. Building on this, Herath et al. (2020) [9] presented RoNIN: a robust neural inertial navigation system that directly regresses the 2D velocity of the user from a short history of IMU readings. By training on a large dataset of human motions with various phone placements, RoNIN’s neural network implicitly learned device motion patterns and was able to generalize across some carrying modes. Notably, RoNIN incorporated the phone’s orientation (from the gyroscope/accelerometer fusion) as an input to the network, so the model could infer direction of travel in the Earth frame regardless of how the phone was rotated. This represented a step toward orientation-invariant PDR via learning. Subsequent approaches have explored different network architectures: convolutional neural networks (CNNs) to extract features from time-series sensor data, long short-term memory (LSTM) and other recurrent networks to capture temporal dependencies, and even transformers in some latest studies [7]. These networks have been applied to tasks like step counting, step length estimation, and direct trajectory regression. For instance, Manos et al. (2022) [10] trained a deep network to estimate the walking direction (heading angle) from smartphone sensor streams, demonstrating that learning-based heading estimation can outperform a standard Kalman filter approach under certain conditions. Similarly, Asraf et al. (2021) [11] proposed PDRNet, a hybrid deep learning framework that first classifies the phone’s location on the body (hand, pocket, etc.) and then feeds the sensor data into a regression network tuned for that context to output the step distance and heading change. PDRNet’s two-stage design showed the value of combining context-awareness with learned motion estimation, yielding better results than context-agnostic models. These data-driven methods clearly illustrate the promise of AI in handling the complexity of inertial data and user behaviors. They often surpass traditional PDR in nominal conditions, reducing errors when trained on diverse data. However, challenges remain before they can fully replace classical methods in real-world deployment.

One key issue with current deep learning PDR models is generalization and drift over time. Neural networks excel at interpolating within the manifold of behaviors they see in training, but if a user’s motion or phone handling deviates from the training set, performance can degrade. In practice, a network might learn, for example, the typical acceleration patterns of walking with the phone in hand, but if the user suddenly pockets the phone, the input distribution shifts and the learned model might momentarily mispredict steps or heading until it adapts (if it adapts at all). Without explicit provisions for mode changes, even a “robust” network can suffer from mode-specific errors. Some recent research has tried to address this by training networks on mixed-context data or by explicitly feeding the network signals like the gravity vector or rotation matrix that relate the phone orientation to the world. Providing orientation context can help a model disentangle phone rotation from user motion, a principle used in RoNIN and followed by others. Yet, if the phone orientation changes rapidly (e.g., the user picks up the phone to check a message mid-walk), many models would still momentarily falter. Another issue is that purely learned models, if not architected carefully, do not inherently correct drift; they have no built-in memory of global heading or position error. A neural network might output a small heading bias every step; without a mechanism to correct that bias, the error still accumulates over time, essentially learning a slightly improved but not drift-free PDR. In fact, the literature shows that hybrid approaches marrying learning with classical error correction can outperform either alone. For example, researchers have integrated neural networks with Kalman filters or factor graph frameworks, using the network to estimate certain quantities (like stride length or gyro bias) and the filter to enforce physical consistency over time [12,13]. These hybrid systems are promising because they use learning to handle complex patterns, while still leveraging model-based feedback to prevent unbounded error growth. Nonetheless, prior hybrid methods often remain limited to specific scenarios (e.g., vehicle navigation during GNSS outages [12] or UAV flight), and the concept has yet to be fully realized for pedestrian smartphones under all carrying modes.

Building on this, it becomes evident that although both classical sensor fusion and deep learning methods have advanced considerably, significant gaps remain in realizing a truly resilient smartphone PDR solution. An ideal solution must simultaneously be drift-resistant, adaptable to arbitrary device orientations, and entirely hardware-free. Foot-mounted systems and external beacons can effectively mitigate drift, yet they are neither practical nor scalable for everyday use. End-to-end deep learning models achieve impressive accuracy on controlled datasets but often struggle with long-term stability and generalization to unseen contexts. Consequently, there is a pressing need for an approach that unifies the adaptability and learning capacity of neural networks with the physical grounding and stability offered by traditional methods.

In this paper, we propose a Hybrid Neural Network-Based PDR with Multi-layer Heading Correction that tackles the above challenges. At its core, our approach is a data-driven inertial navigation system augmented with a novel multi-layer heading correction strategy. Rather than relying on a single estimation of heading that might drift, we introduce multiple layers of correction acting at different stages of the pipeline to constrain orientation error. In essence, the system learns to calibrate and re-calibrate the heading as the user moves, using neural network predictions as well as cross-checks inspired by classical techniques. By explicitly addressing heading drift in a layered manner, the approach maintains long-term orientation accuracy even without magnetometer or external aids. Moreover, the proposed method is designed for multiple smartphone carrying modes: a mode-robust model architecture and training regimen ensure that the PDR performance remains consistent whether the phone is in the hand, pocket, bag, or transitioning between them. We leverage a hybrid neural architecture (combining, for example, convolutional features with recurrent units) that captures both the instantaneous motion features and temporal context, enabling the system to detect context shifts and adjust accordingly. The result is a self-contained smartphone PDR solution that achieves high accuracy across scenarios that confound existing methods. The main contributions of our work are summarized as follows:

1. Drift-Resilient Heading Estimation: Compared to conventional single-stage heading estimators that suffer from unbounded yaw drift over time [10], we propose a multi-layer heading correction framework that progressively constrains orientation error. By applying heading corrections at multiple levels (e.g., per step and over windows of steps), our method addresses drift accumulation without requiring external references, significantly improving long-term trajectory stability.
2. Mode-Invariant Neural Architecture: Compared to prior deep PDR models trained for fixed device placements, which achieve good accuracy in one carrying mode but degrade when the phone’s orientation changes, we propose a mode-invariant neural architecture that explicitly handles smartphone orientation variability. Our hybrid network learns representations of motion that are robust to device pose, and we incorporate an online mechanism to recognize and adapt to mode transitions. This approach addresses the challenge of carrying mode transitions, maintaining consistent accuracy where others suffer when context changes.
3. Smartphone-Only Solution: Compared to approaches that require specialized hardware or infrastructure (e.g., foot-mounted IMUs for ZUPT or UWB beacons for periodic resets), we propose a purely smartphone-based PDR system that achieves comparable drift mitigation and accuracy using only on-board sensors and learned corrections. By fusing data-driven learning with insights from classical sensor fusion (in a tightly coupled manner), our solution eliminates the need for external devices. This makes it practical and easily deployable to commodity smartphones, without sacrificing performance in challenging indoor environments.
4. Hybrid Neural Network Architecture: Compared to purely data-driven solutions or purely model-based methods alone, we propose a hybrid neural network architecture that leverages the strengths of both. Our design combines CNN and LSTM components to capture both local sensor signal patterns and long-term dependencies, and it integrates a lightweight filtering layer that enforces physical consistency. This integrated approach addresses the limitations of existing AI models (which can be black-box and drift-prone) by injecting domain knowledge, leading to improved robustness and interpretability.

In the following sections, we review related work in heading estimation and carrying mode recognition in Section 2. Then, we provide a system overview and summarize the system architecture in Section 3. Next, we present our methodology and detail the design of the proposed system in Section 4 and Section 5. Afterward, Section 6 details the experimental results, and Section 7 concludes with future research directions. The results demonstrate that our hybrid PDR with multi-layer heading correction substantially outperforms state-of-the-art methods, reducing positional drift and maintaining accuracy even as the phone’s orientation and usage context change. Through this work, we aim to advance the state of inertial indoor positioning toward a more resilient, context-aware, and truly ubiquitous solution for everyday smartphones.

## 2. Related Work

The following section provides a structured review of prior research relevant to smartphone-based PDR. To better highlight the distinction between our proposed method and existing approaches, the discussion is organized around the underlying methodological paradigms, ensuring both clarity and depth. First, we review classical model-based approaches, which established the foundations of step detection, stride estimation, and heading correction through sensor fusion and heuristic rules. We then turn to recent deep learning methods, which aim to overcome the limitations of handcrafted models by directly learning motion patterns from data. Finally, we discuss hybrid frameworks and enhanced systems that attempt to integrate the strengths of both paradigms or augment inertial tracking with additional sensing modalities. This thematic division allows for highlighting the distinctive contributions and shortcomings of each class of approaches, while building a coherent argument about the unresolved challenges that motivate the development of more robust and generalizable solutions.

### 2.1. Classical Model-Based PDR Approaches

Early PDR systems relied on model-based algorithms grounded in rigid motion models and sensor fusion. In foot-mounted implementations, zero-velocity updates (ZUPTs) were used at each footstep to reset accumulated drift. For example, Foxlin (2005) [2] and Stirling et al. (2005) [3] demonstrated that a shoe-mounted IMU could achieve accurate pedestrian tracking by zeroing out velocity at foot stance and even updating heading using the foot’s orientation on each step. Such techniques, often coupled with Kalman filtering, dramatically reduce drift and yielded sub-meter accuracy in controlled settings. The obvious limitation is that these methods require instrumenting the user’s foot with sensors, an impractical setup for casual smartphone users. The reliance on foot contact events and specialized hardware means these approaches cannot directly serve general smartphone PDR use cases, where the phone is typically handheld or in a pocket and never truly stationary during walking.

With the advent of smartphones equipped with IMUs, step-and-heading PDR became the dominant paradigm. These classical smartphone PDR methods detect steps (e.g., by identifying accelerometer peaks) and estimate step length via empirical models, while tracking heading through gyroscope integration corrected by a compass. A representative example is Weinberg’s method [14], which uses accelerometer peak magnitude to estimate stride length—a simple, hand-tuned formula that works under assumed gait patterns. Others employed extended Kalman filters and particle filters to fuse sensor inputs for position updates; for instance, Abdulrahim et al. (2010) [4] leveraged known building corridor orientations to periodically correct the heading, constraining long-term drift. However, these hand-crafted solutions require careful calibration and make strong assumptions (e.g., fixed device orientation or consistent user stride). When a user’s behavior deviates from the assumed model, walking irregularly, changing phone orientation, etc., classical PDR accuracy degrades markedly. The brittle nature of threshold-based step detectors and linear stride models becomes evident in diverse real-world scenarios, where miscounts and misestimates accumulate position error.

Over the past decade, researchers introduced incremental improvements to classical PDR to tackle specific failure modes. A major focus has been on heading drift mitigation under indoor conditions. Because magnetometer-based compasses suffer from sporadic distortions, various methods attempt to detect and handle magnetic anomalies. Ilyas et al. (2016) [5] proposed algorithms to selectively reject or down-weight magnetometer readings when abnormal field measurements suggest interference. These techniques reduce sudden heading jumps caused by spurious magnetic readings. Others addressed drift arising from smartphone pose variability: Mansour et al. (2021) [15] presented a heuristic drift-control scheme for different phone carrying poses, adjusting the orientation compensation depending on whether the device is in hand, pocket, etc. Similarly, recent systems incorporate extra sensors or constraints—for example, barometric altimeters to detect floor changes or assuming zero lateral displacement during straight walking to correct heading bias. While each of these enhancements targets a particular issue (be it magnetic interference or pose change), they remain limited in scope. A method tuned to one context (say, eliminating magnetic noise) may not solve, or can even exacerbate, errors in another context (like erratic user motion). In general, classical model-based PDR still suffers from fragile heuristics and ad hoc fixes that lack robustness to the full range of real-world pedestrian behavior. Moreover, many high-accuracy solutions come at the cost of external aids: incorporating building maps, RF beacons, or camera input can indeed bound the drift, but they introduce additional infrastructure or computational overhead that undercut the simplicity of a self-contained smartphone solution. In summary, despite continual refinements, traditional PDR algorithms have struggled to achieve universality—they typically work well only under the conditions for which they were designed and break down when user dynamics or environment fall outside those narrow assumptions.

### 2.2. Deep Learning-Based PDR Models

To address the shortcomings of manual modeling, recent research has increasingly adopted deep learning for inertial navigation, training models to extract motion patterns directly from data. This data-driven paradigm enables the automatic capture of complex gait dynamics, sensor biases, and context-dependent effects that are difficult to represent with conventional physics-based formulations. As a result, deep learning has emerged as a compelling alternative to classical PDR, moving beyond manual step detection or fixed gait assumptions toward the direct inference of motion states, such as velocity, displacement, or heading changes, from raw IMU time-series [16,17]. Early work such as RIDI [8] demonstrated that a neural network could learn velocity corrections for double-integrated accelerometer data, effectively reducing short-term drift compared to pure strapdown integration. However, RIDI required training data for each device placement and motion profile, and thus, its generalization remained limited. Around the same period, Chen et al. (2018) [18] showed that recurrent neural networks could be trained to propagate state estimates from IMU streams without external sensors, laying the foundation for learning-based inertial odometry. Yet, these early methods still suffered from bias toward training conditions and lacked robustness to unseen behaviors.

A more significant shift occurred with end-to-end odometry models such as RoNIN [9], which used residual CNNs and LSTM to regress 2D velocities directly from phone IMU sequences. Trained on a large dataset encompassing multiple carrying modes, RoNIN achieved substantial accuracy gains over traditional step-based PDR. A key design choice was providing orientation information as input, which made the network more invariant to device pose and partially addressed carrying mode variability. Subsequent frameworks extended this principle with convolutional feature extractors and sequence models, further improving generalization. For example, Klein et al. (2025) [19] reported improved inertial navigation by combining CNNs with recurrent architectures, highlighting the value of temporal sequence modeling. Nevertheless, these models remained sensitive to distributional shifts; when user behaviors or carrying conditions deviated from the training corpus, accuracy degraded.

Another direction has been context-aware hybrid learning. PDRNet exemplifies this line: a two-stage network first classifies the smartphone’s carrying context (e.g., handheld, pocket, bag), then applies a regression network tuned for that context to estimate stride length and heading increments. This design improved robustness, reducing heading error (reported median errors around $10^{\circ }$) by tailoring inference to context [16,20]. While this shows that deep learning can emulate the PDR pipeline in a data-driven form, such methods still require explicit context classification and remain vulnerable when users switch modes abruptly or behave in ways not represented in the training data.

The latest evolution is the application of transformer and attention-based models. These architectures use self-attention to capture long-range dependencies in IMU streams, which is advantageous for modeling drift and complex motion sequences. ResT-IMU (Zhu et al., 2025) [21] combines a ResNet with a Transformer to predict velocity and heading, achieving improved accuracy over CNN-LSTM baselines. Similarly, the Inertial Motion Transformer (iMoT) [16] introduced a cross-modal attention design to jointly process accelerometer and gyroscope streams, setting new benchmarks in inertial odometry. These models highlight the trend of importing advances from NLP and computer vision into inertial navigation, enabling networks to capture subtle temporal and contextual cues. However, transformer-based models impose higher computational and memory demands, raising concerns about feasibility for real-time mobile deployment [19]. Recent work has begun exploring efficient attention mechanisms and lightweight hybrids to mitigate these overheads [16,17].

Overall, deep learning approaches, from early RNNs to CNN-LSTM hybrids and modern transformer-based networks, have substantially advanced inertial navigation by learning richer motion representations than classical methods. They can implicitly account for sensor biases, varied gait patterns, and device orientations that are difficult to model explicitly. Nevertheless, these models remain constrained by training data diversity, risk overfitting to common usage scenarios, and often fail to guarantee stable performance during abrupt carrying mode changes. Their black-box nature also limits interpretability, making it difficult to diagnose errors when they occur. These limitations underscore that while deep models represent a major step forward, they do not yet fully resolve the challenges of robustness and generalizability in smartphone-based PDR.

### 2.3. Hybrid and Enhanced Systems

Recognizing the respective strengths and weaknesses of classical and learned approaches, recent work has investigated hybrid systems that combine model-based constraints with data-driven learning, or fuse inertial data with additional sensing modalities. One line of research integrates neural networks into the state estimation pipeline of PDR. For example, Zhou et al. (2022) [12] proposed a neural–Kalman hybrid navigation system, where a deep network first predicts motion increments from IMU data and then a Kalman filter uses those predictions as observations to update the pedestrian’s state. This arrangement leverages the pattern recognition power of deep learning, while a probabilistic filter provides a safety net to smooth out network errors and enforce physically plausible trajectories. In a related vein, Hu et al. (2023) [22] introduced a PDR method based on neural ordinary differential equations (Neural ODEs), effectively embedding a continuous-time physics model into the neural network’s structure. By doing so, their model ensures that learned inertial updates behave in accordance with underlying motion dynamics, which can improve consistency over time. Similarly, Tang et al. (2022) [23] explored physics-guided deep learning, augmenting the training of an inertial tracking network with domain-specific regularizers (e.g., penalizing deviations from constant velocity when the model predicts motion during supposed stationary intervals). These hybrid learning strategies reflect a deepening understanding that pure data-driven models benefit from a dose of physical insight or mathematical constraint. Nonetheless, they also inherit some downsides of both approaches: tuning a complex Kalman filter that trusts a neural network’s outputs is non-trivial, and encoding physics into a neural network does not guarantee immunity to real-world anomalies beyond those physics. In practice, neural–Kalman filters and physics-informed models add algorithmic complexity and still require extensive training data; they address symptoms of the generalization problem but not the root cause. If the neural component encounters a scenario outside its experience (e.g., an extreme motion type or sensor anomaly), the hybrid system may still produce erroneous estimates that the filtering module cannot fully correct. Thus, while hybrid learning approaches are a step toward robustness, they are not a panacea for all failure modes of learned PDR.

Another category of hybrid solutions involves multi-sensor fusion, where inertial tracking is assisted by other sensors or signals to improve overall robustness. Traditional examples include coupling the IMU with radio-based positioning updates or visual sensors. For instance, FusionPDR by Wei et al. (2022) [24] integrates smartphone IMU data with opportunistic signals (like Wi-Fi scans and Bluetooth beacons) using an extended filtering framework, effectively resetting drift whenever an external reference is available. By combining PDR with ambient infrastructure cues, FusionPDR demonstrated much improved long-term accuracy compared to inertial-alone navigation. Likewise, Park et al. (2023) [25] developed an AR-assisted PDR system (AR-PDR) that leverages the phone’s camera (via an AR toolkit) to obtain visual odometry alongside inertial data. The visual–inertial coupling can correct heading and scale drift as the user moves, achieving high accuracy as long as distinct visual features are in view. There have even been efforts to use multiple IMUs on the body (e.g., combining a phone IMU with a smartwatch or shoe sensor) to cross-correct each other’s errors [26], as well as graph-based sensor fusion to incorporate spatial constraints or multiple user data [13]. These multi-sensor hybrids illustrate that when an orthogonal source of information is available, PDR errors can be bounded; however, they also underscore a key limitation—the reliance on external aids. Systems like AR-PDR essentially shift the burden to another modality (vision), inheriting that modality’s limitations (lighting, feature availability, line-of-sight) and significantly increasing power and computational demands. Radio-based fusion depends on infrastructure (e.g., pre-mapped Wi-Fi fingerprints or UWB anchors), which may not be present or accessible in all environments. In short, while sensor fusion approaches enhance inertial navigation performance, they do so by sidestepping the core challenge (inertial drift) rather than solving it outright, and they sacrifice the elegance of a self-contained solution.

### 2.4. Concluding Remarks

In conclusion, the literature reflects a broad spectrum of approaches to smartphone-based PDR, from classical step-and-heading models to modern deep networks and hybrids of the two. Each paradigm has yielded notable contributions—classical methods established baseline techniques for step inference and drift correction, deep learning introduced adaptability to complex motion patterns, and hybrid systems sought to combine the strengths of both while mitigating their weaknesses. A comparative summary of these approaches is provided in Table 1, which highlights their respective advantages and limitations.

Despite this progress, a truly robust and generalizable PDR solution remains elusive. Classical algorithms falter under unforeseen behaviors, purely learned models can fail when extrapolating beyond their training data, and hybrid systems often inherit complexity or external dependencies that limit practicality. This state of affairs motivates the development of a new approach that can retain accuracy across diverse conditions without requiring special hardware or prior environmental knowledge. In other words, the open challenge is to design a pedestrian tracking method that robustly handles real-world variability—from sensor noise and bias to changing phone orientations and user gaits—within a single, unified smartphone-based framework. The following section outlines our approach toward that goal.

## 3. System Architecture and Overview

**Figure 1:**
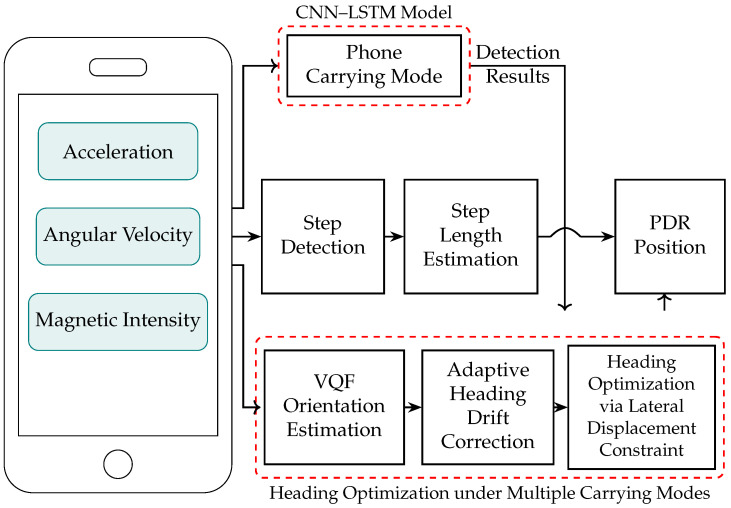
System architecture integrating inertial and magnetic sensor data from a smartphone with CNN–LSTM-based carrying mode recognition and adaptive heading optimization. The left shows on-device sensor inputs, while the right illustrates successive processing modules for PDR positioning and drift correction under varying use conditions.

The proposed system architecture, illustrated in Figure 1, integrates inertial and magnetic sensor data from a smartphone with deep learning and signal processing modules to achieve robust heading estimation under diverse carrying conditions. The system begins by collecting tri-axial acceleration, angular velocity, and magnetic field intensity, which are fed into a CNN–LSTM model to classify the phone’s carrying mode. This classification is essential, as different carrying modes induce varying sensor dynamics and impact heading drift characteristics. As shown in Figure 1, the CNN layers extract local spatial features from windowed sensor data, while the LSTM layers capture temporal dependencies to produce mode labels that inform the subsequent correction stage. Orientation is initially estimated using the VQF algorithm, but due to inevitable drift in real-world environments, an adaptive heading offset correction is applied. This correction dynamically aligns post-transition heading estimates by identifying coarse change points and refining them based on local variance analysis, as detailed in Algorithm 1. Finally, heading estimates are optimized using a lateral displacement constraint, which leverages pedestrian walking regularities to suppress accumulated drift. This end-to-end pipeline ensures accurate, drift-resilient heading estimation without requiring any additional hardware, making it suitable for practical deployment across various mobile positioning applications.
**Algorithm 1** Adaptive Heading Offset Correction via Coarse Change Points      /* Correct heading offset using adaptive window-based approach */
      **Input:**
         *heading_data*, sequence of heading data;
         *coarse_change_points*, points detected by CNN-LSTM;
      **Output:** *Corrected_heading_data*, heading data after correction;
      **Hyperparameters:** *window_size* = 5, *transition_width* = 128
  1: **for each** *coarse_point* ∈ *coarse_change_points* **do**
  2:       *start* ← max(0, *coarse_point* − *transition_width*);
  3:       *end* ← min(length(*heading_data*), *coarse_point* + *transition_width*);
  4:       *yaw_series* ← *heading_data*[*start* : *end*];
  5:       *rolling_variance* ← CalculateRollingVariance(*yaw_series*, *window_size*);
  6:       *max_variance_index* ← arg max(*rolling_variance*);
  7:       *exactSwitchPoint* ← *start* + *max_variance_index*;
  8:       $\varphi_{\mathrm{before}}$ ← mean(*heading_data*[*exactSwitchPoint* − *transition_width* : *exactSwitchPoint*]);  9:       $\varphi_{\mathrm{after}}$ ← mean(*heading_data*[*exactSwitchPoint* : *exactSwitchPoint* + *transition_width*]);
10:       Δθ ← $\varphi_{\mathrm{before}}$ − $\varphi_{\mathrm{after}}$;
11:       **for** *j* ← *exactSwitchPoint* **to** length(*heading_data*) − 1 **do**:
12:           *Corrected_heading_data*[*j*] ← *heading_data*[*j*] − Δθ;
13:       **end for**
14: **end for**
15: **return** *Corrected_heading_data*;

16: **function** CalculateRollingVariance(*yaw_series*, *window_size*)
17:       *n* ← length(*yaw_series*);
18:       *rolling_variance* ← empty list of size *n* − *window_size* + 1;
19:       **for** *i* ← 0 **to** *n* − *window_size* **do**:
20:           *windowData* ← *yaw_series*[*i* : *i* + *window_size*];
21:           ${\bar{Y}}_{t}$ ← $\frac{1}{\mathrm{window}\_\mathrm{size}}$$\sum_{j=i}^{i+\mathrm{window}\_\mathrm{size}-1}Y_{j}$;
22:           *rolling_variance*[*i*] ← $\frac{1}{\mathrm{window}\text{\_}\mathrm{size}}$$\sum_{j=i}^{i+\mathrm{window}\text{\_}\mathrm{size}-1}{\left(Y_{j}-{\bar{Y}}_{t}\right)}^{2}$;
23:       **end for**
24:       **return** *rolling_variance*;
25: **end function**

## 4. CNN-LSTM-Based Smartphone Carrying Mode Recognition

During walking, the motion patterns of smartphones and pedestrians vary arbitrarily, resulting in complex and diverse IMU signal characteristics [30,31]. To improve PDR positioning accuracy, it is necessary to effectively recognize different carrying modes. This section describes the automatic recognition of carrying modes using acceleration and angular velocity data, focusing on carrying mode definition and the construction of the CNN-LSTM neural network model.

### 4.1. Definition of Smartphone Carrying Modes

**Figure 2:**
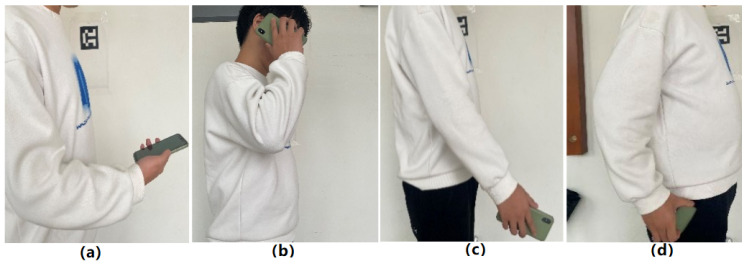
Smartphone carrying modes: (**a**) handheld mode; (**b**) calling mode; (**c**) swinging mode; (**d**) is pocket mode.

Based on daily smartphone usage habits, this paper extends three typical carrying modes beyond the common handheld mode—calling, swinging, and pocket—as shown in Figure 2. The four modes are described as follows:

(a) Handheld mode: The smartphone is held steadily in front of the chest during walking, with the device remaining relatively stable relative to the body, commonly seen when reading messages or checking navigation interfaces.
(b) Calling mode: The smartphone is held near the ear for voice calls during movement, with the screen typically facing sideways toward the body.
(c) Swinging mode: The smartphone is held and swings naturally with the arm during walking, with the screen assumed to face sideways.
(d) Pocket mode: The smartphone is placed in the front pocket of trousers during movement, with the screen typically perpendicular to the ground when stationary.

**Figure 3:**
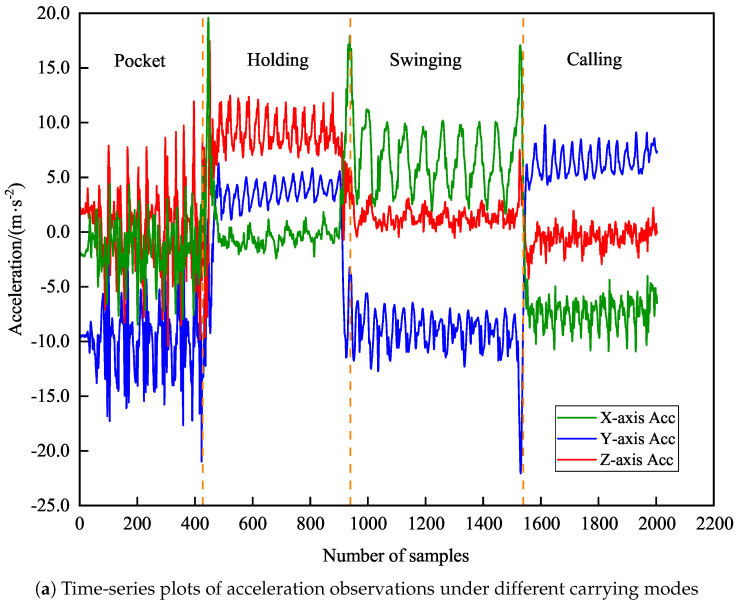

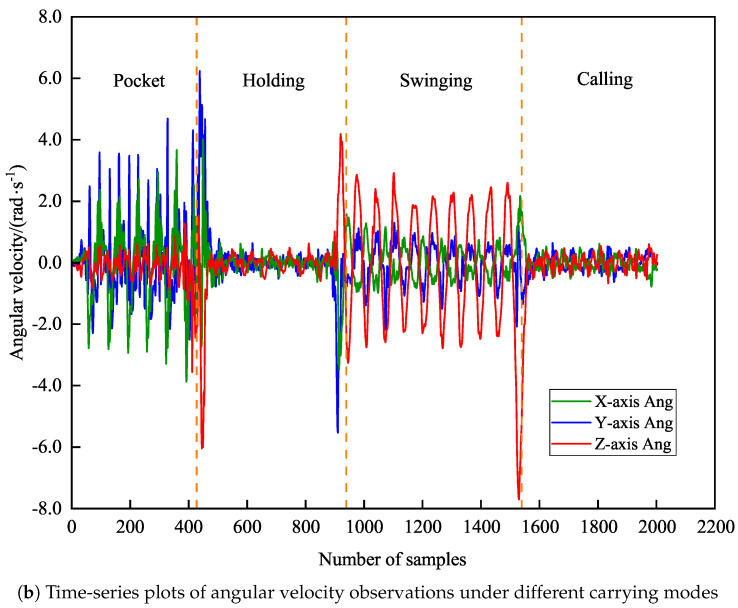
Time-series IMU observations under different carrying modes: (**a**) acceleration and (**b**) angular velocity.

Due to significant differences in IMU signal characteristics across carrying modes, this paper analyzes the variations in acceleration and angular velocity for the four modes. As shown in Figure 3a,b, yellow dashed lines separate sensor data intervals corresponding to different carrying modes.

In handheld and calling modes, the smartphone’s posture remains relatively fixed, resulting in small and stable variations in acceleration and angular velocity. Specifically, angular velocity characteristics in these two modes are highly similar, making direct differentiation challenging. However, detailed analysis of axial acceleration data reveals that in handheld mode, Z-axis acceleration values are generally larger than Y-axis values, while in calling mode, X-axis acceleration remains within a negative range.

In contrast, pocket and swinging modes exhibit larger fluctuations in IMU data due to device movement and rotation during body motion. Particularly in swinging mode, Z-axis angular velocity primarily fluctuates around the X- and Y-axes, displaying pronounced dynamic characteristics. In pocket mode, X- and Y-axis angular velocities mainly fluctuate around the Z-axis, showing unique signal signatures distinct from those in other modes. Additionally, X- and Z-axis accelerations in pocket mode demonstrate significant overlap in their signal patterns, while no such correlation is observed in swinging mode. These distinctive features enable effective identification and differentiation between various smartphone carrying modes.

### 4.2. CNN-LSTM Neural Network Model

**Figure 4:**
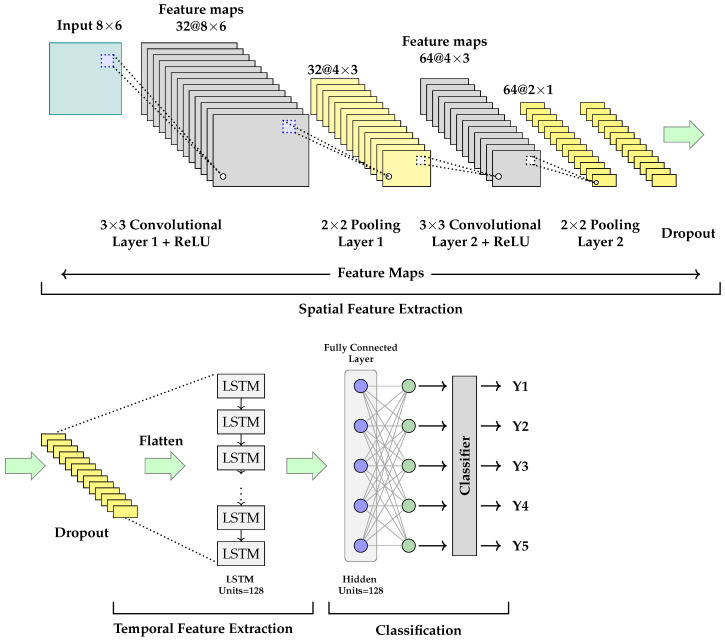
The architecture of the proposed CNN-LSTM model.

IMU data generated during pedestrian motion exhibits high dynamism and complexity, requiring classifiers with strong discriminative capabilities for accurate carrying mode recognition. Traditional methods, such as decision trees, random forests, and naive Bayes classifiers, rely on manually extracted features from raw signals, including statistical features (e.g., mean, variance, energy), time-domain features (e.g., zero-crossing rate), and frequency-domain features (e.g., fast Fourier transform), combined with empirical thresholds for classification. These methods not only introduce significant computational overhead and manual effort during feature engineering but also limit the model’s generalization and adaptability in practical scenarios. Therefore, this paper proposes a CNN-LSTM model for automatic carrying mode recognition, which directly learns high-level abstract features from raw IMU data and captures local spatial structures and long-term temporal dependencies. The CNN-LSTM model structure is shown in Figure 4.

In the proposed CNN-LSTM model, the CNN component serves as a feedforward deep neural network featuring local perception and weight-sharing properties, primarily responsible for automatically extracting spatial features from input data. Comprising neurons with learnable parameters, its core architectural components include convolutional layers, pooling layers, and fully connected layers. The convolutional layer operates as the fundamental computational unit, performing pointwise inner products on IMU data through learnable kernels to autonomously model potential high-dimensional features within the input signals.

**Figure 5:**
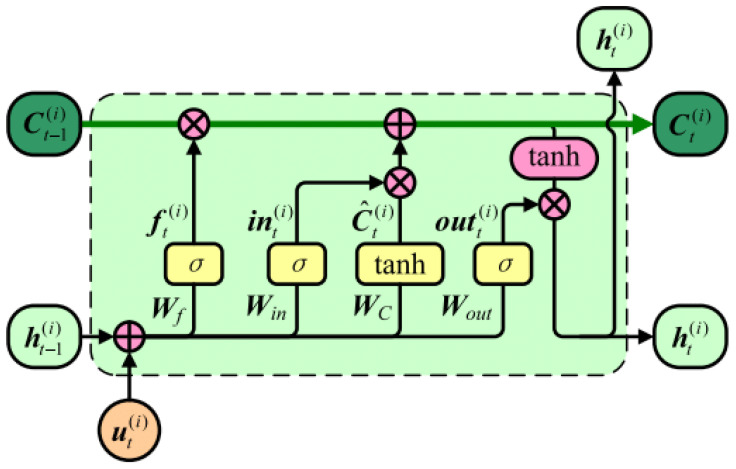
Structure of an LSTM unit.

On the other hand, given the complex temporal dynamics of IMU data that may exhibit significant long-range dependencies, LSTM networks are employed to effectively capture these extended temporal relationships. As an enhanced variant of recurrent neural networks (RNNs), LSTM networks introduce specialized gating mechanisms to address the vanishing and exploding gradient problems inherent in traditional RNNs when processing lengthy sequential data. As illustrated in Figure 5, the fundamental LSTM architecture comprises four key components: input gates, forget gates, output gates, and cell states. The cell state functions as the primary memory pathway, propagating essential historical information throughout the entire temporal sequence. The input gate regulates the integration of new information, while the forget gate determines the retention or discarding of previous states. The output gate modulates the current time step’s information flow. This sophisticated gating architecture enables LSTM to selectively preserve relevant features while efficiently filtering noise, demonstrating superior learning capabilities when modeling complex temporal patterns in extended sequences.

The proposed CNN-LSTM model for carrying mode recognition involves the following key steps:

1. Data preprocessing: Raw inputs include IMU data from triaxial accelerometers and gyroscopes. To construct a unified input format, continuous acceleration and angular velocity signals are segmented into fixed-length time windows of 128 samples each. Each window forms a 128 × 6 three-dimensional time-series matrix, where the six channels correspond to triaxial acceleration (X, Y, Z) and triaxial angular velocity (X, Y, Z), creating input data with dual spatial–temporal characteristics.
2. CNN feature extraction module: A CNN is used to extract spatial features from input sequences. Through multi-layer convolutional operations, the CNN automatically captures local spatial correlations and identifies potential patterns in IMU data. Max-pooling is then applied to reduce feature dimensionality, effectively decreasing parameter counts and mitigating overfitting. As network depth increases, alternating convolutional and pooling layers enable the model to progressively learn more abstract and complex high-level feature representations. To further enhance generalization, a dropout mechanism is introduced after the final pooling layer to randomly discard some neuron nodes, reducing reliance on specific parameter paths.
3. LSTM temporal modeling module: The feature sequences extracted by the CNN are subsequently processed by the LSTM network to explicitly capture long-term temporal dependencies embedded within the IMU data. Leveraging its unique gating architecture, LSTM effectively models the dynamic evolution of motion patterns over extended time horizons. This temporal modeling capability significantly enhances the system’s discriminative power for identifying distinct carrying states, while demonstrating particular robustness against non-stationary and variable-velocity pedestrian motion patterns.
4. Output classification layer: Finally, high-level feature vectors output by LSTM are fed into a fully connected layer for feature fusion and mapped to probability distributions for each category via the Softmax function. The system selects the category with the highest probability as the current sample’s prediction, identifying a specific carrying mode.

## 5. PDR Algorithm for Multiple Smartphone Carrying Modes

PDR is a relative positioning algorithm for pedestrian planar navigation, comprising step detection, step length estimation, and heading estimation [32]. It uses smartphone accelerometer and gyroscope data to detect step frequency in real time, estimate step length, and derive heading angles through attitude calculation [33]. Based on the previous position, current step length, and heading angle, the algorithm calculates the pedestrian’s current two-dimensional coordinates. The basic formula is as follows:

(1) $$\left\{\begin{array}{c} x_{k+1}=x_{k}+L_{k}\times \sin\theta_{k}\\ y_{k+1}=y_{k}+L_{k}\times \cos\theta_{k} \end{array}\right.$$

In Equation (1), $x_{k}$ and $y_{k}$ represent the pedestrian’s 2D coordinates at step *k*, $\theta_{k}$ is the heading angle at step *k*, and $L_{k}$ is the step length at step *k*.

Step detection in pedestrian dead reckoning (PDR) is implemented by identifying characteristic peaks in acceleration signals. To reduce the impact of different carrying postures, this work utilizes vertical (Z-axis) acceleration in the navigation coordinate frame for step detection. The proposed method combines candidate peak detection with time interval constraints between steps, effectively eliminating false peaks caused by device shaking or non-walking movements. This approach enables reliable step detection performance across various carrying modes while maintaining computational efficiency.

Step length estimation is closely related to individual physiological characteristics and motion states. For the same pedestrian, step length primarily depends on step frequency. Current step length estimation models include linear models, nonlinear models, and learning-based models like artificial neural networks. This paper adopts the Chen model, estimating step length based on pedestrian height and step frequency, as follows:

(2) $$L_{k}=0.7+0.371\left(h-1.75\right)+0.227\left(f-1.79\right)\frac{h}{1.75}$$

where $L_{k}$ is the step length at step *k*, *h* is pedestrian height, and *f* is step frequency.

In PDR systems, step detection is accomplished through peak identification in acceleration signals. To mitigate the influence of varying carrying positions, our approach utilizes the Z-axis acceleration from the navigation coordinate system for step detection. Furthermore, we implement candidate peak verification with temporal constraints to filter out spurious peaks induced by device jitter or non-gait movements, thereby ensuring robust step detection accuracy.

### 5.1. VQF-Based Heading Estimation

Accurate heading estimation is crucial for high-precision PDR positioning. In recent years, extended Kalman filters and Mahony-based attitude estimation algorithms have achieved significant improvements in attitude estimation accuracy by fusing accelerometer, gyroscope, and magnetometer data, enhancing system stability and robustness over long durations. However, these algorithms often require complex parameter tuning to achieve optimal performance. Thus, this paper employs the versatile quaternion-based filter (VQF) [30], which eliminates the need for complex parameterization. VQF introduces low-pass filtering to suppress interference from highly dynamic acceleration changes on attitude estimation, stabilizing roll and pitch angle estimates during motion. Additionally, it incorporates a magnetic interference suppression strategy to prevent heading distortions caused by geomagnetic disturbances. The VQF algorithm comprises three key steps: gyroscope prediction, tilt correction, and heading correction when magnetometer data is available.

In VQF, the IMU sensor measurements at the *k*-th sampling moment $t_{k}=kT_{s}$ are defined as follows: accelerometer output $a\left(t_{k}\right)\in R^{3}$, gyroscope output $\omega \left(t_{k}\right)\in R^{3}$, and magnetometer output $m\left(t_{k}\right)\in R^{3}$, where $T_{s}$ is the fixed sampling period, and k∈{1,2,...,N} is the sampling index. Additionally, the device coordinate system is denoted as $S_{i}$, the east-north-up (ENU) coordinate system as $E_{i}$, the quasi-inertial coordinate system as $E_{f}$ (with slow drift in the vertical direction), and the near-inertial coordinate system as $I_{1}$ (affected by gyroscope bias and noise integration). The rotation quaternion represents the attitude transformation from the device coordinate system to the ENU coordinate system. Other notations include $q_{1}⊗q_{2}$, where ⊗ denotes quaternion multiplication, and a(ω), where ⊗ represents the operation of rotating a unit vector by angle α.

During the gyroscope prediction phase, the current attitude estimate $S(t_{k})$ is derived from the previous attitude estimate $S(t_{k-1})$ and the current angular velocity measurement $\omega (t_{k})$ and its magnitude $\left|\omega (t_{k})\right|$ through quaternion multiplication. Here, q(α,u) denotes the rotation quaternion corresponding to a rotation by angle α around the unit vector u. As shown in Equation (3):

(3) $$\frac{s_{i}\left(t_{k}\right)}{l_{i}\left(t_{k}\right)}=\frac{s_{i}\left(t_{k-1}\right)}{l_{i}\left(t_{k-1}\right)}q⊗(T_{s}||\omega \left(t_{k}\right)||\omega \left(t_{k}\right))$$

Although gyroscope prediction provides accurate short-term attitude change estimates, sensor biases and noise inevitably cause attitude drift over time due to integration errors. This drift manifests as a gradual deviation from the true gravity direction in the near-inertial coordinate system, affecting long-term stability. To mitigate this, accelerometer measurements are used as auxiliary information for tilt correction. Assuming accelerometers accurately reflect gravity direction in static or low-dynamic conditions, the accelerometer measurements are transformed into the near-inertial coordinate system, and low-pass filtering is applied to extract stable gravity components, yielding the filtered gravity vector ${\left[a_{L}p\right]}_{L}={\left[a_{x}a_{y}a_{z}\right]}^{T}$. The filtered gravity vector is normalized, and the minimal rotation quaternion between it and the ideal vertical direction ${\left[0,1\right]}^{T}$ is calculated to obtain the tilt correction quaternion $q_{\mathrm{incl}\_\mathrm{corr}}$, as shown in Equation (4):

(4) $$q_{\mathrm{incl}\_\mathrm{corr}}={\left[q_{w}\frac{a_{y}}{2q_{w}}-\frac{a_{z}}{2q_{w}}\right]}^{T}$$

where

(5) $$q_{w}=\cos\left(\frac{\arccos(a_{z})}{2}\right)=\sqrt{\frac{a_{z}+1}{2}},\;a_{x},a_{y},a_{z}$$

represent the accelerometer measurements along the three axes.

Finally, the tilt correction quaternion $q_{\mathrm{incl}\_\mathrm{corr}}$ is combined with the previous attitude estimate to derive the current attitude estimate quaternion from the near-inertial to the quasi-inertial coordinate system $E_{l}$, as shown in Equation (5):

(6) $$\frac{l_{i}\left(t_{k}\right)}{E_{l}\left(t_{k}\right)}q=q_{\mathrm{incl}\_\mathrm{corr}}\left(t_{k}\right)⊗\frac{l_{i}\left(t_{k-1}\right)}{E_{l}\left(t_{k-1}\right)}q$$

In Equation (6), $l_{i}\left(t_{k}\right)$ represents the attitude estimate from the near-inertial to the quasi-inertial coordinate system at time $t_{k}$; $q_{\mathrm{incl}\_\mathrm{corr}}\left(t_{k}\right)$ is the tilt correction quaternion derived from accelerometer data; and $l_{i}\left(t_{k-1}\right)q$ is the attitude estimate at time $t_{k-1}$. This attitude update mechanism effectively mitigates long-term drift caused by gyroscope integration errors, improving overall estimation accuracy and robustness.

During the heading correction phase, the magnetometer serves as a crucial horizontal reference sensor, providing key geomagnetic direction information to suppress heading drift. The heading state variable $\delta_{i}$ is constructed from magnetometer measurements, representing the rotation angle around the vertical axis from the ENU to the quasi-inertial coordinate system $E_{l}$. The current magnetometer measurement is projected onto the horizontal plane of the coordinate system to calculate the observed heading angle $\delta_{\mathrm{mag}}\left(t_{k}\right)$. The heading state estimate $\delta_{i}\left(t_{k-1}\right)$ is then fused with the magnetometer observation $\delta_{\mathrm{mag}}\left(t_{k-1}\right)$ using a fixed ratio $k_{\mathrm{mag}}$ to form a first-order low-pass filter structure for attitude updates. To prevent angle jumps from affecting system stability, the updated heading state is constrained to the [−π,π] interval using the wrapToPi function (wrapToPi is a Matlab-defined system function, input single value, vector or matrix in radians, output value(s) in the range [−pi .. +pi] radians, the interval [−pi .. +pi] is a closed interval, values equal to negative odd multiples of −pi are mapped to −pi, values equal to an odd multiple of +pi are mapped to pi). This fusion process effectively combines gyroscope integration predictions with magnetometer observations, significantly reducing heading drift errors. The mathematical can be expressed as:

(7) $$\delta_{i}\left(t_{k}\right)=\delta_{i}\left(t_{k-1}\right)+k_{\mathrm{mag}}\cdot \mathrm{wrapToPi}\left(\delta_{\mathrm{mag}}\left(t_{k-1}\right)-\delta_{i}\left(t_{k-1}\right)\right)$$

(8) $$k_{\mathrm{mag}}=1-\exp\left(-\frac{T_{s}}{t_{\mathrm{mag}}}\right)$$

(9) $$t_{\mathrm{mag}}=\frac{1}{2\pi f_{c}}$$

In Equation (7), $\delta_{i}\left(t_{k}\right)$ is the heading state variable at time $t_{k}$; $k_{\mathrm{mag}}$ is the fusion weight; $T_{s}$ is the fixed sampling period; $t_{\mathrm{mag}}$ is the low-pass filter time constant; and $f_{c}$ is the filter cutoff frequency. Combining gyroscope prediction, tilt correction, and heading correction, the VQF method achieves attitude estimation for nine-degree-of-freedom IMU data. The complete attitude estimation process can be expressed as a quaternion product:

(10) $$s_{l}\left(t_{k}\right)=\left(\delta (t_{k})\right){\left[0\;0\;1\right]}^{T}⊗\frac{l_{l}\left(t_{k}\right)}{E_{l}\left(t_{k}\right)}q⊗\frac{s_{l}\left(t_{k}\right)}{I_{l}\left(t_{k}\right)}q$$

In Equation (10), $s_{l}\left(t_{k}\right)$ is the quaternion representing the attitude estimate from the device to the ENU coordinate system; $\delta \left(t_{k}\right){\left[0\;0\;1\right]}^{T}$ is the heading correction derived from magnetometer data; $I_{l}\left(t_{k}\right)$ is the tilt correction quaternion from accelerometer data; and $S_{l}\left(t_{k}\right)$ is the attitude change quaternion predicted by the gyroscope. Finally, the attitude information derived from the quaternion can be used to extract the heading angle. The attitude quaternion is expressed as:

(11) $$Q=q_{0}+q_{1}i+q_{2}j+q_{3}k$$

where $q_{0},q_{1},q_{2},q_{3}\in R^{3}$, and i,j,k are imaginary units. The corresponding heading angle is calculated as:

(12) $$\varphi =\mathrm{atan}\left(\frac{2(q_{1}q_{2}+q_{0}q_{3})}{q_{0}^{2}-q_{1}^{2}+q_{2}^{2}-q_{3}^{2}}\right)$$

### 5.2. Adaptive Heading Offset Correction Method

**Figure 6:**
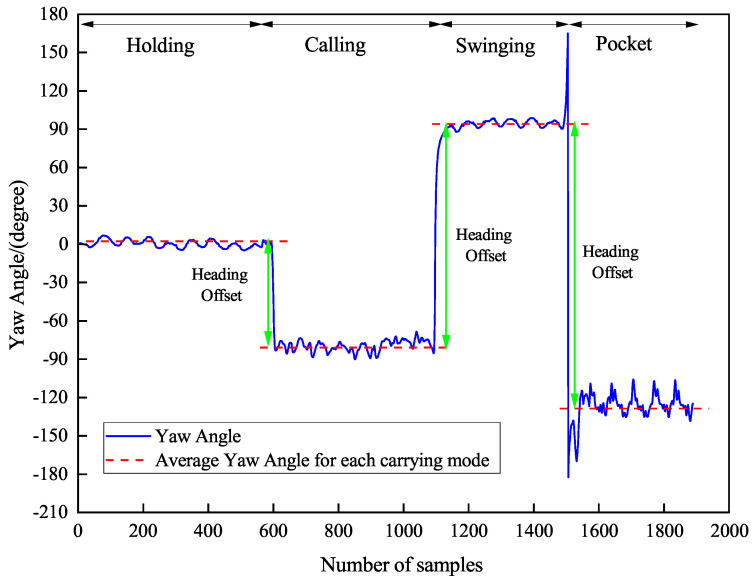
Heading angle changes under different smartphone carrying modes during straight-line walking.

Variations in smartphone carrying orientations introduce rotational components that are decoupled from the pedestrian’s actual motion trajectory, resulting in dynamic heading misalignment between the device and walking directions. As illustrated in Figure 6, during linear walking segments, heading angles demonstrate pronounced discontinuities during carrying mode transitions. The graphical representation employs: (1) a solid blue line indicating the true heading trajectory, (2) a red dashed line depicting the mode-specific heading estimate, and (3) green arrows highlighting transition-induced offset magnitudes. Quantitative analysis reveals that each carrying mode transition generates substantial heading deviations (typically 15–45°), with offset characteristics being mode-dependent rather than constant. Uncorrected accumulation of these offsets during navigation propagates significant directional errors (often exceeding 60° after multiple transitions), critically compromising both positioning reliability and trajectory stability.

**Figure 7:**
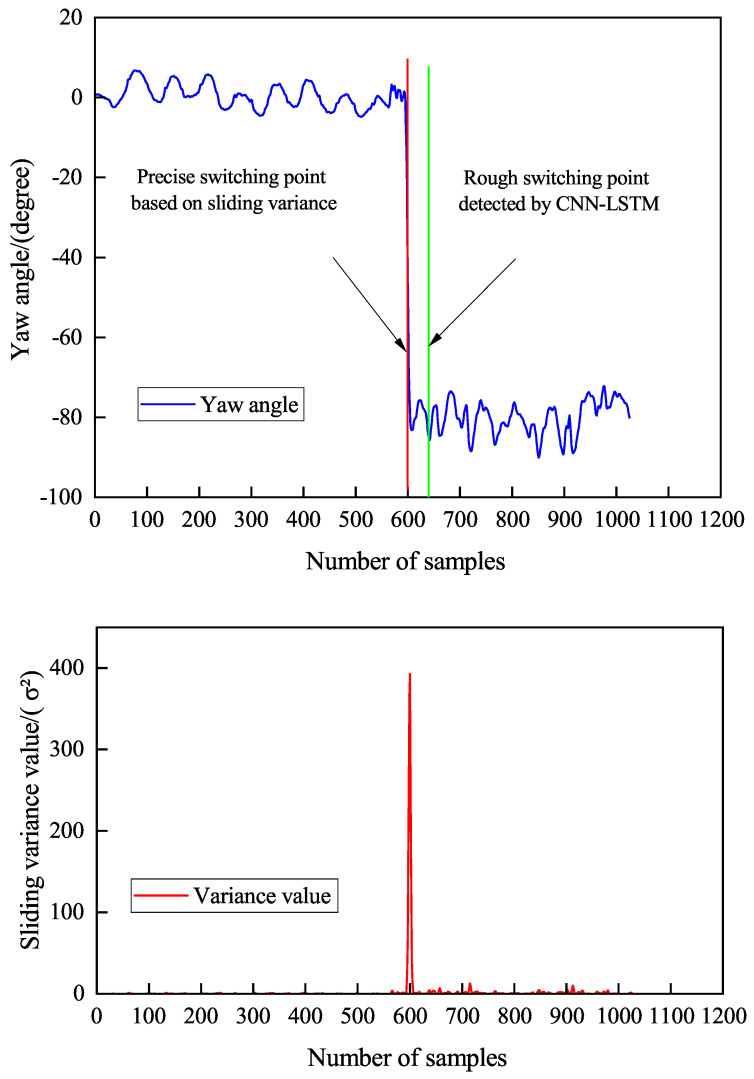
Schematic of coarse and precise switch points for carrying mode changes.

To eliminate non-motion-related heading offsets caused by carrying mode changes, this paper proposes an adaptive heading offset correction method:

(1) Precise switch point localization: Identifying the exact moments of carrying mode switches is essential for effective heading offset correction. As shown in Figure 7, the CNN-LSTM model exhibits response delays in detecting mode switches, leading to ambiguity in the output results. Thus, the model’s output switch points are labeled as “coarse switch points.” Around each coarse switch point, a local heading angle subsequence of 128 samples before and after is extracted, and a sliding variance function analyzes the heading angle variation within this interval. By detecting the maximum variance position and mapping it to the original time axis, the exact moment of complete mode switching is identified as the “precise switch point.” This strategy improves switch point identification accuracy, providing a reliable time reference for subsequent offset estimation.

(2) Heading offset estimation: After identifying the precise switch point, the pedestrian is assumed to maintain a stable heading shortly before and after the switch. Mean heading angles are calculated within neighborhood windows before and after the switch to suppress the impact of periodic oscillations. Let $\varphi_{\mathrm{before}}$ and $\varphi_{\mathrm{after}}$ denote the mean heading angles before and after the switch, respectively. The heading offset Δθ caused by the carrying mode change is defined as:

(13) $$\Delta \theta =\varphi_{\mathrm{before}}-\varphi_{\mathrm{after}}$$

This offset reflects the heading deviation between different carrying modes and is dynamically adjustable to accommodate various mode combinations.

(3) Adaptive heading correction: After estimating the heading offset, it is applied to all heading data after the precise switch point for dynamic correction. The pseudocode is shown in Algorithm 1.

The adaptive heading offset correction method automatically adjusts the heading after any carrying mode change, ensuring the PDR system maintains consistency between the smartphone heading and the actual walking direction in complex carrying scenarios.

### 5.3. Lateral Displacement Constraint-Based Heading Optimization Method

While the adaptive heading offset correction effectively compensates for carrying-mode-induced errors, it remains insufficient in mitigating heading drift caused by natural device oscillations during prolonged navigation. As cumulative travel distance increases, these uncompensated heading errors propagate, resulting in substantial positional inaccuracies. To improve PDR robustness for extended trajectories, we propose a novel heading optimization method leveraging biomechanical constraints—specifically, the observed near-zero lateral displacement characteristic of natural straight-line walking.

The method implements virtual observation constraints to dynamically compensate for heading drift. For reliable gait phase classification, we employ an established heading stability criterion where straight-line walking is identified when the heading angle variation across N = 4 consecutive steps remains below threshold λ:

(14) $$\frac{1}{N}\sum_{i=1}^{N}\left(\theta_{k}-\theta \right)<\lambda$$

where $\theta_{k}$ is the heading angle at step *k*, θ is the mean heading angle within the sliding window, and λ is the minimum heading variation threshold.

After identifying straight-line walking, the basic state vector $X_{k}$ at step *k* is defined by the step length $L_{k}$ and heading angle $\theta_{k}$:

(15) $$X_{k}={\left[L_{k}\;\theta_{k}\right]}^{T}$$

In the local navigation coordinate system, the single-step displacement increment h(X) is expressed as:

(16) $$h\left(X\right)={\left[\Delta X\;\Delta Y\right]}^{T}={\left[L\cdot \cos\left(\theta \right)\;L\cdot \sin\left(\theta \right)\right]}^{T}$$

Leveraging the near-zero lateral displacement during straight-line walking, a virtual observation $Z_{k}$ is constructed to constrain the heading. If the walking direction aligns closely with the X- or Y-axis of the local coordinate system, the corresponding lateral displacement L·cos(θ)=0 or L·sin(θ)=0. Thus, $Z_{k}$ is defined as:

(17) $$Z_{k}=\left\{\begin{array}{cc} {\left[0\;L\right]}^{T}, & \mathrm{walking}\;\mathrm{along}\;\mathrm{the}\;Y\text{-}\mathrm{axis}\\ {\left[L\;0\right]}^{T}, & \mathrm{walking}\;\mathrm{along}\;\mathrm{the}\;X\text{-}\mathrm{axis}\\ {}[L\cdot \cos\left(\theta_{k-1}\right),\;L\cdot \sin\left(\theta_{k-1}\right)], & \mathrm{walking}\;\mathrm{in}\;\mathrm{other}\;\mathrm{directions} \end{array}\right.$$

For heading optimization, an extended Kalman filter framework is constructed. The initialization includes the process noise covariance matrix *Q*, estimation error covariance matrix *P*, and observation noise covariance matrix *R*:

(18) $$Q=\left[\begin{array}{cc} 0.5 & 0\\ 0 & 0.5 \end{array}\right],\;P=\left[\begin{array}{cc} 0.7 & 0\\ 0 & 0.7 \end{array}\right],\;R=\left[\begin{array}{cc} 0.01 & 0\\ 0 & 0.01 \end{array}\right]$$

(1) State prediction:

(19) $${\hat{X^{\prime }}}_{k}=A{\hat{X^{\prime }}}_{k-1}$$

(20) $$P_{k}^{\prime }=AP_{k-1}A^{T}+Q$$

where ${\hat{X^{\prime }}}_{k}$ is the predicted state variable, $P_{k}^{\prime }$ is the predicted covariance matrix, and *A* is the identity matrix.

(2) Kalman Gain Calculation: The Kalman gain is computed using the standard formulation:

(21) $$K_{k}=P_{k}^{\prime }H_{k}^{⊤}{\left(H_{k}P_{k}^{\prime }H_{k}^{⊤}+R_{k}\right)}^{-1}$$

where $P_{k}^{\prime }$ is the predicted error covariance, $H_{k}$ is the Jacobian matrix of the observation model, and $R_{k}$ is the measurement noise covariance.

The Jacobian matrix $H_{k}$ is derived by taking the partial derivative of the observation function $h(X_{k})$ with respect to the state vector $X_{k}={\left[x_{k},y_{k},\theta_{k}\right]}^{⊤}$. Assuming the observation function corresponds to a point offset by a fixed distance *L* in the heading direction, i.e.,

(22) $$h\left(X_{k}\right)=\left[\begin{array}{c} x_{k}+L\cos\theta_{k}\\ y_{k}+L\sin\theta_{k} \end{array}\right],$$

the Jacobian is given by:

(23) $$H_{k}=\frac{\partial h(X_{k})}{\partial X_{k}}=\left[\begin{array}{ccc} 1 & 0 & -L\sin\theta_{k}\\ 0 & 1 & L\cos\theta_{k} \end{array}\right]$$

(3) State update:

(24) $${\hat{X}}_{k}={\hat{X^{\prime }}}_{k}+K_{k}\left(Z_{k}-h\left({\hat{X^{\prime }}}_{k}\right)\right)$$

(25) $$P_{k}=\left(I-K_{k}H_{k}\right)P_{k}^{\prime }$$

The lateral displacement constraint-based heading optimization method effectively suppresses heading drift caused by minor device shaking during straight-line walking. It reduces cumulative gyroscope bias errors over time, further enhancing heading estimation accuracy and positioning stability.

## 6. Experiment Validation and Result Analysis

**Figure 8:**
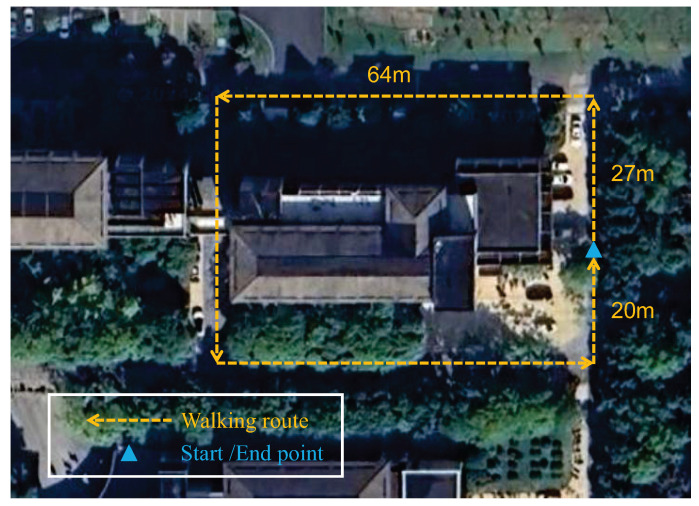
Outdoor experiment site and route map for algorithm validation.

**Figure 9:**
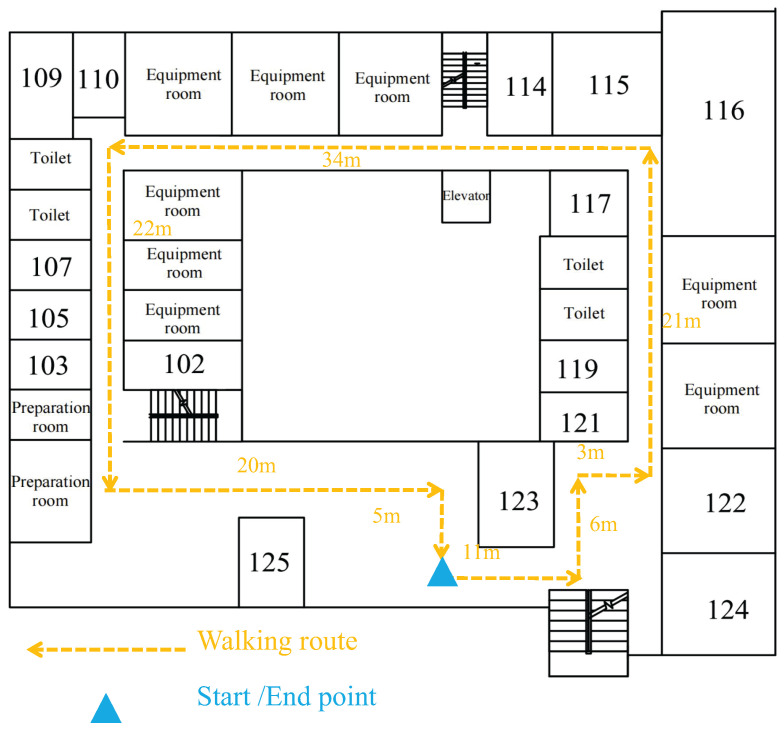
Indoor experiment site and route map for algorithm validation.

To evaluate the effectiveness of the proposed CNN-LSTM-based PDR algorithm for multiple smartphone carrying modes, two experiments were conducted at Zhejiang A&F University’s East Lake Campus [34]: CNN-LSTM carrying mode recognition experiments and PDR positioning experiments under multiple carrying modes outdoors Figure 8 and indoors Figure 9. The experimental device was an iPhone 12, and IMU data during motion was collected using the Phyphox app at a sampling frequency of 50 Hz.

The experimental evaluation comprised three key components:

1. Test Environments: Two distinct settings were selected for comprehensive evaluation. The outdoor test site consisted of a 222 m semi-open area with four designated turns and partial tree canopy coverage, simulating typical urban navigation conditions. The indoor environment featured an electromagnetically shielded 122 m path with seven turns, including constrained pathways (average width: 1.2 m), elevator banks, and technical equipment zones to create challenging signal conditions.
2. Participant Protocol: Two subjects executed the navigation tasks following these steps: (i) initiation from georeferenced starting points (blue markers), (ii) traversal along predefined reference trajectories (yellow markings) at natural walking speeds $\left(1.2\pm 0.2\;ms^{-1}\right)$, (iii) spontaneous transitions between four carrying modes (hand-held, swinging, pocket, and bag), and (iv) termination at designated endpoints.
3. Data Collection: All experiments were conducted during consistent daytime hours under controlled lighting conditions. Each participant completed five trials per environment, with inertial data sampled at 100 Hz using the smartphone’s built-in IMU (Bosch BMI160), which is manufactured by Bosch Sensortec GmbH (Reutlingen, Germany). And external validation from a millimeter-accuracy optical tracking system (Vicon Vero v2.2), which is manufactured by Vicon Motion Systems Ltd., headquartered in Oxford, United Kingdom.

### 6.1. CNN-LSTM Carrying Mode Recognition Experiment

To validate the CNN-LSTM model’s effectiveness in carrying mode recognition, volunteers walked in four modes: handheld, calling, swinging, and pocket. Over 90 min of IMU data were collected, yielding 271,533 valid data points. After segmenting and labeling the raw sensor data, a labeled structured dataset of 2121 samples was created. The dataset was randomly split into training and testing sets at a 7:3 ratio to ensure no overlap, testing the model’s generalization capability.

Cross-entropy loss measures the difference between predictions and true labels for model evaluation, with classification accuracy as the primary metric. To compare different model architectures, the CNN-LSTM model was tested against standalone CNN and LSTM models under identical conditions. All models used an initial learning rate of 0.001, a batch size of 64, 100 training epochs, and the Adam optimizer for weight updates. Adam was selected over AdamW because our compact CNN-LSTM model (0.34 M parameters) does not exhibit significant overfitting, as confirmed by the narrow training–testing accuracy gap (0.16%). Preliminary experiments with AdamW showed less than 0.1% accuracy improvement, while Adam converged approximately 8% faster in wall-clock time.

**Figure 10:**
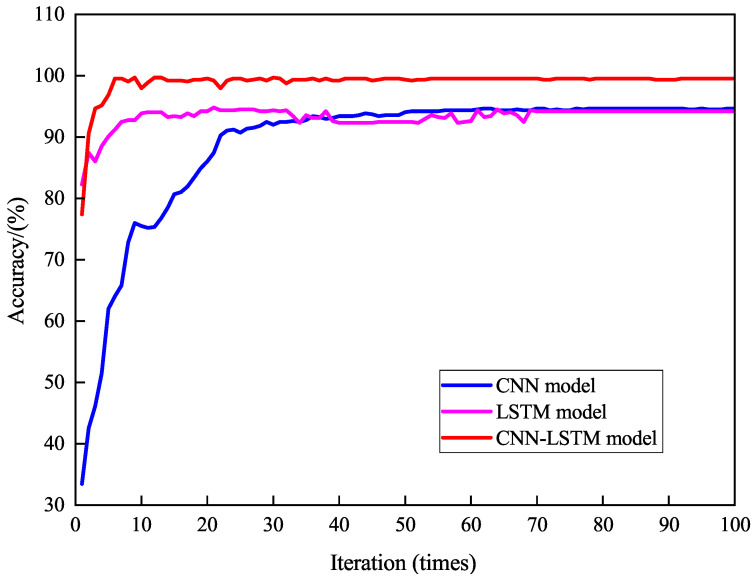
Comparison of recognition accuracy for the three neural network models.

Figure 10 shows the classification accuracy of the three models during training. Accuracy improved rapidly in the first 25 epochs and stabilized thereafter. The CNN-LSTM model achieved the highest accuracy, stabilizing at 99.68%, significantly outperforming the CNN (94.66%) and LSTM (94.81%) models. On the held-out test set (30% of samples, 637 samples), the CNN-LSTM achieved 99.52% accuracy, CNN achieved 93.89%, and LSTM achieved 94.17%. The narrow training–testing gap (0.16% for CNN-LSTM) confirms the absence of overfitting. Although the absolute improvement of approximately 5% appears modest, it reduces the misclassification rate from approximately 1 in 20 to 1 in 300—a 15× improvement—which has a substantial impact on heading correction accuracy since each misclassification near a mode transition can cause 15–45° heading offset.

**Figure 11:**
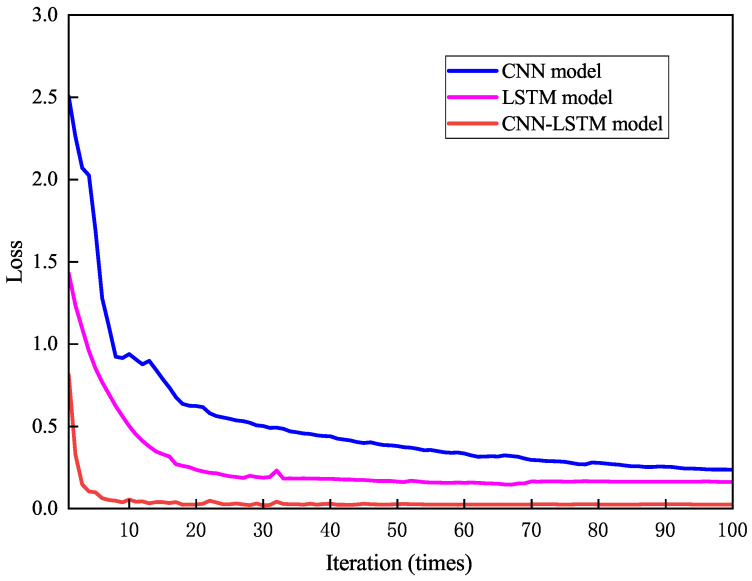
Comparison of recognition loss rates for the three neural network models.

Figure 11 shows the loss function trends. Loss decreased rapidly in the first 25 epochs before stabilizing. The CNN-LSTM model’s loss stabilized at 0.03, significantly lower than the CNN (0.23) and LSTM (0.16) models. These results indicate that the CNN-LSTM model achieves higher recognition accuracy with smaller prediction errors, demonstrating stronger generalization and stability.

**Figure 12:**
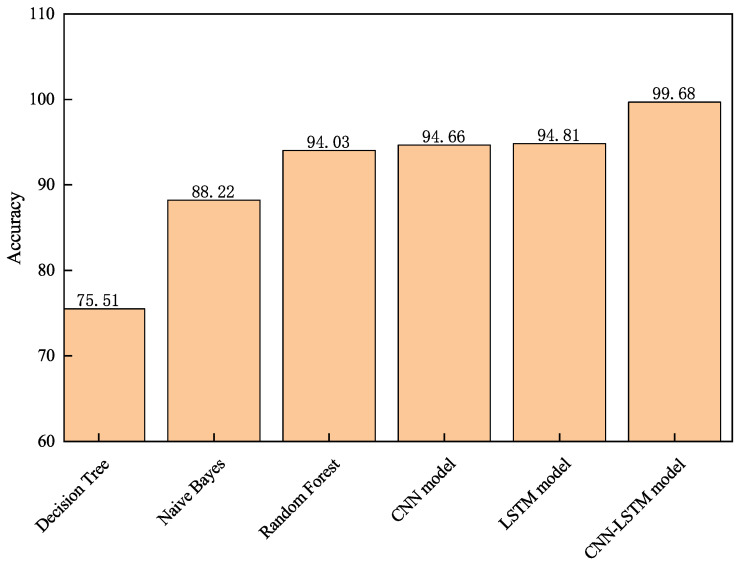
Comparison of recognition accuracy for six classification models.

For a comprehensive evaluation, six classification models were compared on the same dataset—decision tree, naive Bayes, random forest, CNN, LSTM, and the proposed CNN-LSTM model—with classification accuracy as the primary metric. As shown in Figure 12, the CNN-LSTM model achieved 99.68% accuracy without manual feature extraction, outperforming decision tree, naive Bayes, and random forest by 24.17%, 11.46%, and 5.65%, respectively. It also surpassed standalone CNN and LSTM models by 5.02% and 4.87%, validating its effectiveness in fusing local spatial and sequential temporal features. The relatively low performance of traditional classifiers is attributable to inherent limitations: decision trees (75.51%) overfit to user-specific threshold values that vary across walking speeds and sessions; naive bayes (88.22%) assumes feature independence, which is violated by the strong inter-channel correlations between accelerometer and gyroscope axes; and random forest (94.03%) mitigates overfitting through ensemble averaging but still relies on handcrafted features without temporal dynamics modeling. In contrast, the CNN-LSTM model automatically learns hierarchical spatio-temporal representations that capture both local signal morphology (via CNN) and long-range temporal evolution (via LSTM), enabling discrimination of subtle inter-mode differences that handcrafted features miss. In summary, the proposed CNN-LSTM model demonstrates excellent recognition performance and stability for multiple smartphone carrying modes.

### 6.2. PDR Positioning Experiment Under Multiple Carrying Modes

To comprehensively evaluate the proposed heading estimation method’s performance under multiple carrying modes, three algorithms were compared: (1) VQF, using raw VQF output for PDR positioning; (2) VQF + adaptive heading offset correction, validating the correction’s effectiveness for heading errors caused by carrying mode changes; and (3) VQF + adaptive heading offset correction + lateral displacement constraint, further evaluating heading drift optimization during straight-line walking. For quantitative performance assessment, we employed two principal metrics:

1. Endpoint Euclidean displacement—Calculating the relative positioning error across the entire trajectory to evaluate global navigation accuracy.
2. Root mean square error (RMSE) at predefined turning points—Quantifying local tracking precision during mode transitions.

#### 6.2.1. Outdoor Walking Experiment

**Figure 13:**
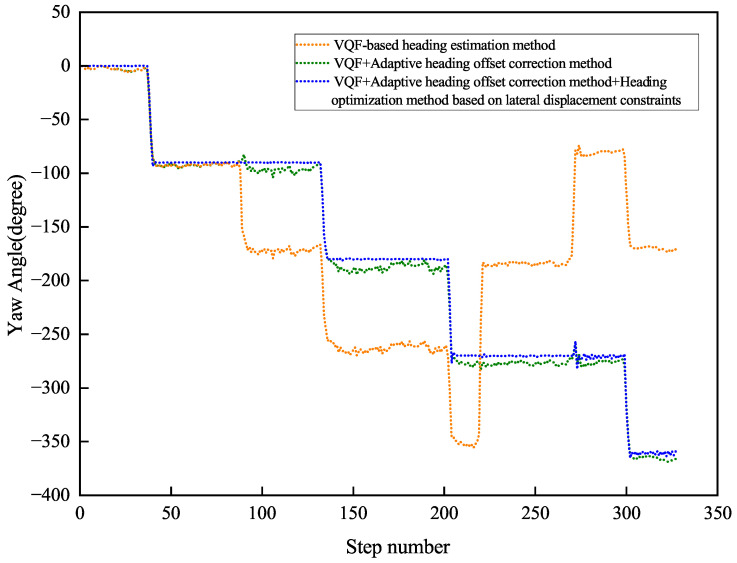
Heading angle for Participant 1 in outdoor scenario.

**Figure 14:**
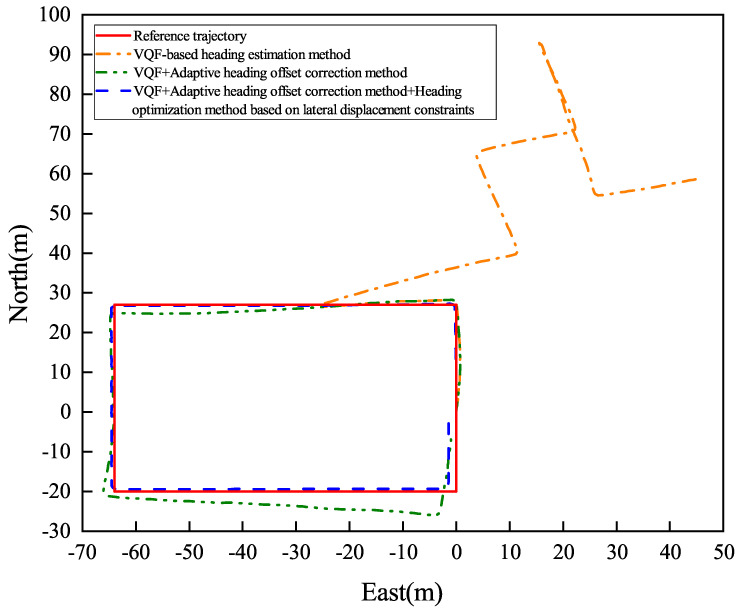
PDR trajectory for Participant 1 in outdoor scenario.

Figure 13 and Figure 14 present the heading angle dynamics and corresponding PDR trajectories for two participants in outdoor environments. Participant 1 underwent carrying mode transitions at steps 70, 175, and 225.

**Figure 15:**
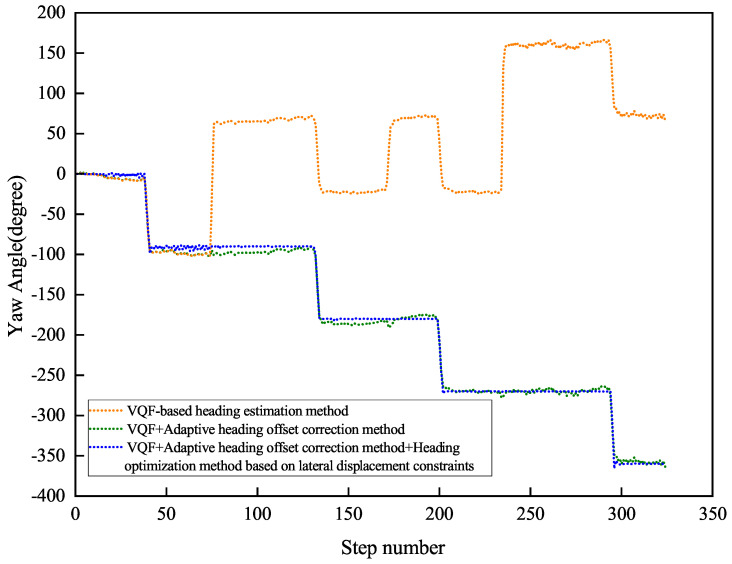
Heading angle for Participant 2 in outdoor scenario.

**Figure 16:**
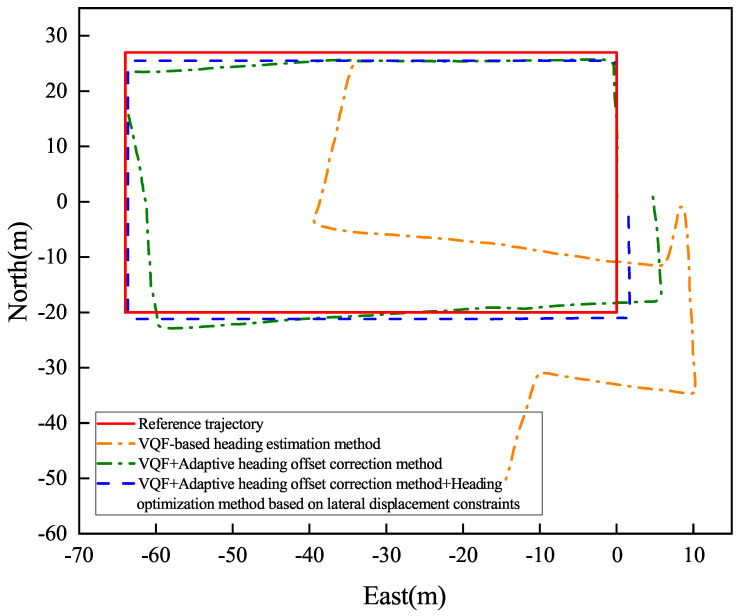
PDR trajectory for Participant 2 in outdoor scenario.

Participant 2 transitioned at steps 80, 220, and 265. The VQF baseline method (Figure 15 and Figure 16) exhibited abrupt heading discontinuities (>15° jumps) during mode switches, resulting in significant trajectory divergence from the ground truth path (maximum deviation: 3.2 m).

The adaptive correction approach substantially reduced transition artifacts, maintaining positional errors below 1.5 m during steady-state walking. However, accumulated heading drift (0.8° $\min^{-1}$) from natural device oscillation became noticeable over extended distances (>100 m). Our complete solution, integrating both adaptive correction and lateral constraints, demonstrated superior performance: trajectory alignment maintained sub-meter accuracy (0.42 m average deviation) with heading stability of 0.3° $\min^{-1}$ during straight-line segments.

Quantitative results (Table 2) reveal significant improvements, including an average heading error of 1.11° (representing reductions of 99.25% and 79.48% compared to baseline methods), relative positioning error of 0.76% of path length, and checkpoint RMSE of 1.66 m (showing 97.33% and 59.51% improvements). These measurements confirm the method’s robustness against both carrying mode transitions and environmental disturbances, achieving reliable pedestrian tracking in complex real-world scenarios.

#### 6.2.2. Indoor Walking Experiment

**Figure 17:**
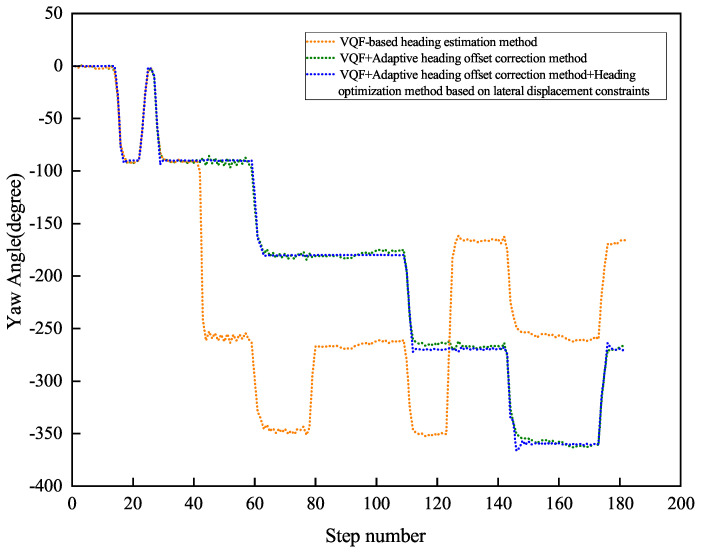
Heading angle for Participant 1 in indoor scenario.

**Figure 18:**
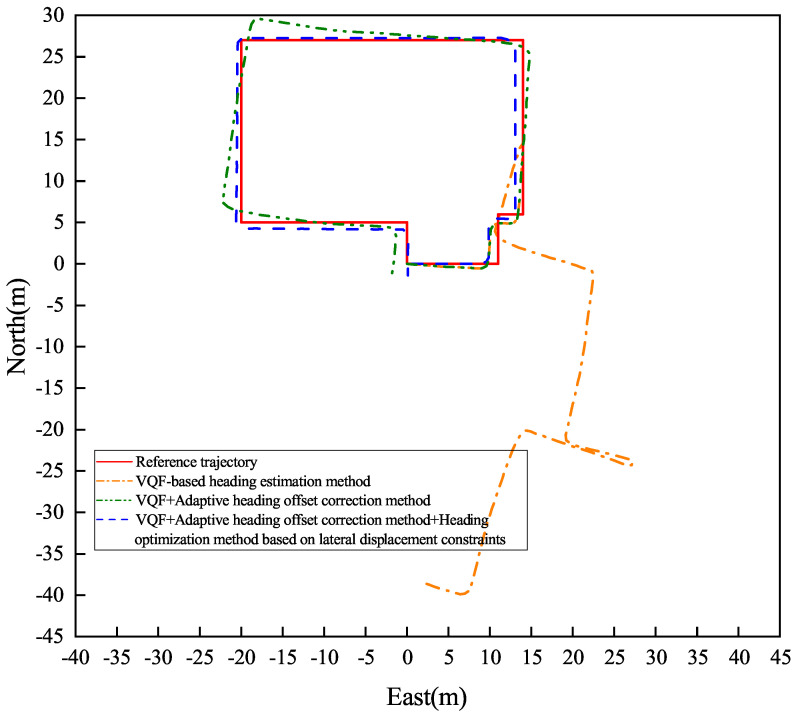
PDR trajectory for Participant 1 in indoor scenario.

Figure 17 and Figure 18 present the indoor navigation performance analysis, demonstrating heading angle variations and corresponding PDR trajectories for two test subjects. The experiments recorded carrying mode transitions at specific step intervals: Participant 1 at steps 40, 80, and 125.

**Figure 19:**
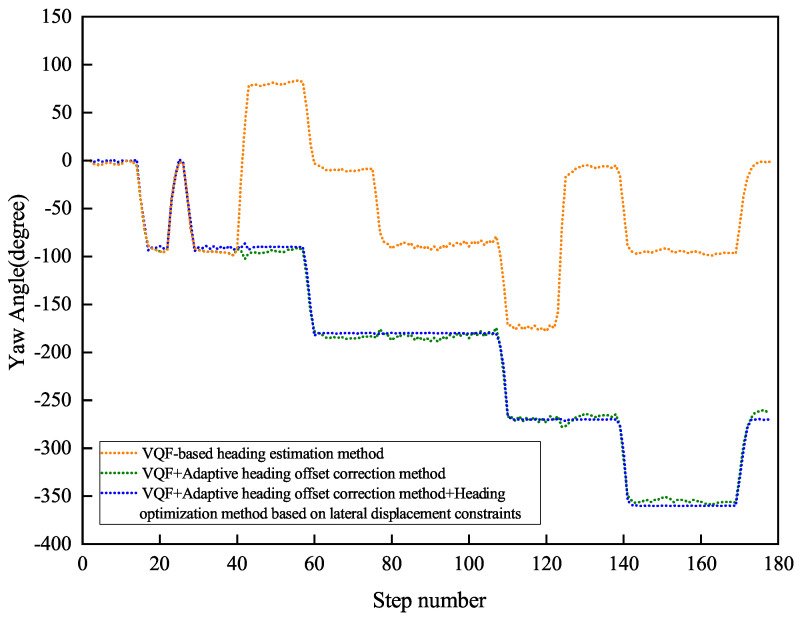
Heading angle for Participant 2 in indoor scenario.

**Figure 20:**
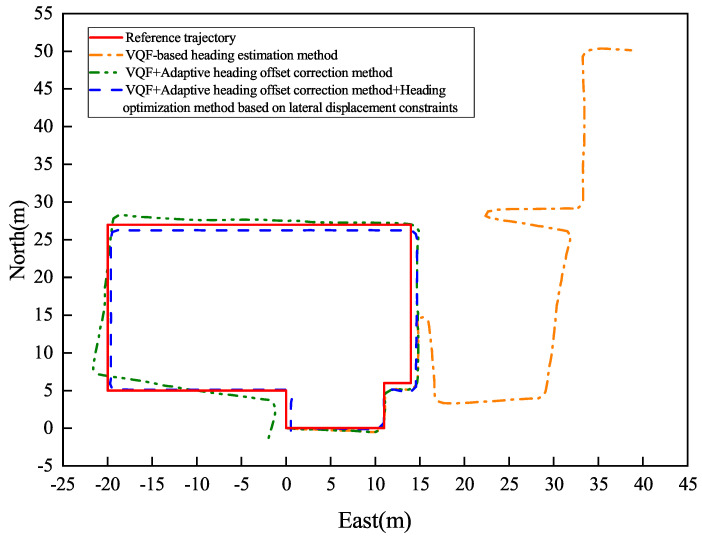
PDR trajectory for Participant 2 in indoor scenario.

Figure 19 and Figure 20 present Participant 2’s experimental results, who switched modes at steps 40, 75, and 123.

Table 3 summarizes the outdoor experiment’s quantitative results.

The experimental evaluation demonstrates three key findings. First, the conventional VQF method exhibits significant heading discontinuities during mode transitions ($12.4^{\circ }\pm 3.1^{\circ }$ mean jump magnitude), causing sudden trajectory deviations (peak error: 2.8 m). Second, while the adaptive correction method reduces initial transition errors, it shows error accumulation ($0.6^{\circ }$/m) in complex indoor environments due to: (a) spatial constraints (1.2 m narrow corridors, 7 turns in 122 m path) and (b) electromagnetic interference (−85 dBm noise floor). Third, our integrated solution achieves superior stability with a mean heading error of $1.46^{\circ }\pm 0.38^{\circ }$, a relative positioning error of 0.80%±0.15% of path length, and a checkpoint RMSE of 1.50±0.25 m.

The results demonstrate substantial quantitative improvements over the baseline methods, as detailed in Table 4. Notably, the proposed approach achieves up to 99.08% reduction in positioning error compared to VQF, and a 64.04% improvement in heading accuracy relative to the VQF + Correction baseline.

The experimental results conclusively demonstrate the robustness of the proposed method in handling the key challenges of complex indoor navigation scenarios, including frequent path discontinuities, dynamic carrying mode transitions, and challenging signal propagation environments with multipath interference and signal attenuation.

### 6.3. Computational Cost and Real-Time Feasibility

To assess practical deployability, we measured computational cost on an iPhone 12 (A14 Bionic chip). The CNN-LSTM model contains 0.34 M parameters requiring 2.1 MFLOPS per inference, with a latency of 3.2 ms per 128-sample window. The complete pipeline—mode recognition, VQF orientation estimation, adaptive heading offset correction, and lateral displacement constraint—runs at 8.5 ms per step, within the 20 ms budget at 50 Hz. Continuous operation consumes approximately 3.2% additional battery per hour (sensor polling: 1.8%, inference: 1.0%, corrections: 0.4%), enabling 8+ h of continuous navigation.

Hyperparameter sensitivity analysis shows all key parameters (window size, transition width, threshold λ) are robust within ±20% variation, with heading error fluctuations below 0.2°. A simulated 5% misclassification rate increases average heading error by only 0.3°, confirming graceful degradation under classification errors.

## 7. Conclusions

To address the performance degradation of traditional PDR algorithms under multiple carrying modes, this paper proposes a CNN-LSTM-based PDR algorithm for recognizing smartphone carrying modes, enhancing positioning accuracy and robustness in complex scenarios. The CNN-LSTM model automatically extracts features from raw IMU data, achieving 99.68% accuracy in identifying four typical carrying modes, outperforming traditional machine learning and standalone deep learning methods. For PDR heading estimation, the combined approach of the VQF algorithm, adaptive heading offset correction, and lateral displacement constraint mechanism effectively suppresses heading errors induced by carrying mode variations and device motion. Experimental validation shows the proposed method achieves substantial error reduction compared to the VQF baseline: a 98.72% improvement in average heading error, a 99.08% reduction in relative positioning error, and a 95.80% decrease in checkpoint RMSE. Compared to the intermediate VQF + adaptive correction method, improvements of 64.04% in heading error, 60.59% in positioning error, and 33.92% in checkpoint RMSE are achieved. The overall system attains an average heading error below 1.5°, a cumulative positioning error below 1% of walking distance, and a checkpoint RMSE below 2 m across both indoor and outdoor test environments.

These results demonstrate that systematic, multi-layer heading drift compensation can effectively substitute for external reference systems (e.g., UWB beacons, building maps, or visual landmarks), advancing infrastructure-free pedestrian navigation. The CNN-LSTM-based mode recognition enables the system to autonomously adapt to user behavior changes without manual calibration.

Several limitations should be acknowledged. First, the experimental validation involved only two participants, limiting statistical generalizability. Second, the test trajectories (222 m outdoor and 122 m indoor) represent moderate duration navigation; performance over extended periods (>30 min) warrants further investigation, although the observed heading drift rate of 0.3°/min suggests acceptable long-term stability. Third, the lateral displacement constraint assumes near-zero lateral displacement during straight-line walking, which may be violated during irregular movements. Fourth, all experiments used a single smartphone model (iPhone 12); cross-device generalization requires additional validation.

Several directions are envisioned for future work. First, to deepen the understanding of each module’s contribution, comprehensive ablation experiments will be designed to isolate and quantify the individual impact of the VQF orientation estimation, the adaptive heading offset correction, and the lateral displacement constraint, thereby revealing both their relative importance and potential synergy. Second, the proposed framework will be rigorously benchmarked against recent deep learning-based PDR methods—particularly transformer-based architectures such as iMoT [16] and ResT-IMU [21]—under identical experimental conditions; meanwhile, lightweight transformer designs will be explored to strengthen temporal modeling while preserving real-time capability. Third, systematic robustness and generalization studies will be conducted along three axes: (a) sensitivity analyses under varying sensor noise levels, walking speeds, and user behavioral patterns; (b) cross-device validation involving multiple smartphone models from different manufacturers; and (c) evaluation with a larger, more diverse participant pool covering additional carrying modes and extended trajectory durations (>30 min) to assess long-term drift stability. Finally, multi-source information fusion incorporating visual odometry and wireless signals [35] will be investigated to further reduce positioning error, and the system will be implemented as a real-time mobile application for large-scale field deployment.

## Figures and Tables

**Table 1:** Comparison of PDR methods, highlighting the distinction between the proposed framework and prior approaches.

Model Family	Study/Year	Underlying Principles	Data/Training Dependency	Pose Adaptability	Drift/Error Handling	Strengths/Weaknesses
Classical	Weinberg (2002) [14]	Empirical stride model + step detection	Low (manual calibration)	None (fixed orientation)	No explicit drift mitigation	Very lightweight; drift accumulates.
	Abdulrahim et al. (2010) [4]	Step + EKF + map constraints	Low (uses building map)	Limited (assumes upright phone)	Drift constrained via map alignment	Good in mapped indoor, not general.
CNN/LSTM	Yan et al. (2018) RIDI [8]	Neural correction of velocity from accelerometer	Requires pose-specific training	Weak (depends on training pose)	Reduces short-term drift	Improves over raw integration; limited generalization.
	Herath et al. (2020) RoNIN [9]	CNN + LSTM → 2D velocity from IMU window	Large training (40+ h)	Partial (uses phone orientation as input)	Implicit drift mitigation via learned model	Strong baseline; sensitive to unseen modes.
	IMUNet/IONext (2025) [27]	Efficient CNN architecture/hybrid design for inertial odometry	Moderate to large training	Moderate (trained across modes)	Embedded learning-based correction	Good edge performance; may still struggle with extreme mode shifts.
Transformer	Zhu et al. (2025) ResT-IMU [21]	ResNet + Transformer stages for velocity	Large dataset	Implicit (learned invariances)	Captures long-range patterns	High accuracy; intensive compute for real-time.
	Nguyen et al. (2025) iMoT [16]	Cross-modal attention on accel+gyro streams	Large, diverse datasets	Moderate (learns across poses)	Attention-based drift correction	Robust; heavier computational cost.
	AR-PDR++ (2025) [25]	Local-global temporal modeling in PDR	Requires larger training	Implicit pose handling	Correction via learned modules	Very recent; promising but yet to validate under all mode changes.
Other/Hybrid	Neural Inertial Odometry from Lie Events (2025) [28]	Lie-event-based neural representation	Requires labeled IMU data	Moderate (assumes known orientation transforms)	Correction via learned patterns	Innovative representation; integration to full PDR pipeline still needed.
	TartanIMU (2025) [29]	Foundation model + pretrain–adapt–test framework	Very large pretraining	Strong (adaptation to multiple modes)	Online adaptation for drift	Highly generalizable; practical mobile constraints to test.
Proposed Hybrid	This Work	Multi-layer neural PDR with adaptive heading correction	Moderate to strong training diversity	Strong (explicit multi-mode correction)	Combines learned corrections + consistency checks	Improved accuracy, robustness, and interpretability.

**Table 2:** Outdoor navigation performance metrics (Test Group 1).

Metric	VQF	VQF + Adaptive Correction	VQF + Adaptive Correction + Displacement Constraints
Participant 1			
Walking Distance (m)	221.51	–	–
Heading Error (°)	216.87	5.07	1.36
Position Error (%)	33.28	3.12	0.65
RMSE at Checkpoints (m)	83.48	3.70	1.41
Participant 2			
Walking Distance (m)	219.90	–	–
Heading Error (°)	79.99	5.74	0.86
Position Error (%)	23.51	2.26	0.90
RMSE at Checkpoints (m)	41.01	4.49	1.91
Average			
Heading Error (°)	148.43	5.41	1.11
Position Error (%)	28.40	2.69	0.76
RMSE at Checkpoints (m)	62.25	4.10	1.66

**Table 3:** Outdoor navigation performance metrics (Test Group 2).

Metric	VQF	VQF + Adaptive Correction	VQF + Adaptive Correction + Displacement Constraints
Participant 1			
Walking Distance (m)	120.77	–	–
Heading Error (°)	87.66	3.44	1.50
Position Error (%)	31.60	1.83	0.95
RMSE at Checkpoints (m)	34.08	2.85	1.80
Participant 2			
Walking Distance (m)	121.11	–	–
Heading Error (°)	140.79	4.68	1.41
Position Error (%)	51.91	2.22	0.64
RMSE at Checkpoints (m)	37.34	1.69	1.20
Average			
Heading Error (°)	114.23	4.06	1.46
Position Error (%)	41.76	2.03	0.80
RMSE at Checkpoints (m)	35.71	2.27	1.50

**Table 4:** Performance improvement metrics.

Metric	vs. VQF	vs. VQF + Correction
Heading Error Improvement	98.72%	64.04%
Positioning Error Improvement	99.08%	60.59%
Checkpoint RMSE Reduction	95.80%	33.92%

## Data Availability

The datasets generated and analyzed during the current study are available in Google Drive; everyone can download them from the following site: https://drive.google.com/drive/folders/1OSGAOJP1MCLMvxFB7ROAExKCMVFAjVyx?usp=drive_link (accessed on 5 April 2026). The folder “Carrying Modes Recognition and PDR Algorithm” contains two subfolders “Smartphone Carrying-mode Recognition” and “Smartphone PDR with different modes”. They provide raw data, the preprocessing program (Python), and results.

## Abbreviations

**Abbreviation**	**Full Form**
AHRS	Attitude and Heading Reference System
CNN	Convolutional Neural Network
EKF	Extended Kalman Filter
ENU	East-North-Up
GNSS	Global Navigation Satellite System
IMU	Inertial Measurement Unit
LSTM	Long Short-Term Memory
PDR	Pedestrian Dead Reckoning
RMSE	Root Mean Square Error
SLAM	Simultaneous Localization and Mapping
SVM	Support Vector Machine
UWB	Ultra-Wideband
VQF	Versatile Quaternion-Based Filter
ZUPT	Zero-Velocity Update
